# Effect of a Nurse Navigation Intervention on Mental Symptoms in Patients With Psychological Vulnerability and Breast Cancer: The REBECCA Randomized Clinical Trial

**DOI:** 10.1001/jamanetworkopen.2023.19591

**Published:** 2023-06-23

**Authors:** Pernille Envold Bidstrup, Christoffer Johansen, Niels Kroman, Federica Belmonte, Helle Duriaud, Susanne Oksbjerg Dalton, Kenneth Geving Andersen, Birgitte Mertz

**Affiliations:** 1 Psychological Aspects of Cancer, Danish Cancer Society Research Center, Copenhagen, Denmark; 2 Department of Psychology, University of Copenhagen, Copenhagen, Denmark; 3 Late Effect Research Unit, CASTLE, Oncology Clinic, University Hospital Rigshospitalet, Copenhagen, Denmark; 4 Danish Cancer Society, Copenhagen, Denmark; 5 Department of Breast Surgery, Herlev/Gentofte Hospital, Copenhagen, Denmark; 6 Statistics and Data Analysis, Danish Cancer Society Research Center, Copenhagen, Denmark; 7 Survivorship and Inequality in Cancer, Danish Cancer Society Research Center, Copenhagen, Denmark; 8 Department of Clinical Oncology and Palliative Care, Zealand University Hospital, Næstved, Denmark; 9 Department of Anesthesiology, Hvidovre Hospital, Copenhagen, Denmark; 10 Section for Surgical Pathophysiology, Rigshospitalet, University of Copenhagen, Copenhagen, Denmark

## Abstract

**Importance:**

The unmet needs regarding symptom management of psychological distress among patients with breast cancer must be addressed. However, little evidence exists on effective interventions, such as nurse navigation.

**Objective:**

To compare the long-term effects of the REBECCA (Rehabilitation After Breast Cancer) nurse navigation intervention vs usual care in patients with breast cancer who were psychologically vulnerable.

**Design, Setting, and Participants:**

This parallel randomized clinical trial recruited and evaluated for eligibility adult female patients with newly diagnosed breast cancer and symptoms of psychological distress (distress score of ≥7 points on Distress Thermometer) at Rigshospitalet in Copenhagen, Denmark, from August 2017 to October 2019. This study continued the work of a pilot study, extending the follow-up to 18 months. Patients who met the inclusion criteria were randomized to either standard care or the REBECCA intervention. Intention-to-treat analyses were performed from June 2021 to October 2022.

**Interventions:**

Patients who were randomized to the REBECCA intervention received nurse navigation and symptom screening as well as standard care. Standard care included regular treatment, nurse support at chemotherapy and radiotherapy appointments, and municipality-based rehabilitation.

**Main Outcomes and Measures:**

The primary outcome was distress, as measured using the Distress Thermometer. The secondary outcomes included symptoms of anxiety, symptoms of depression, breast cancer–specific health-related quality of life, fear of recurrence, sleep, cognitive function, patient activation, pain, health behavior, body mass index, and need for support. Long-term effects at 6, 12, and 18 months were examined using mixed-effect models, adjusting for randomization strata of age and treatment modality.

**Results:**

A total of 309 female patients were included in the analysis, with 153 patients randomized to the standard care group and 156 patients randomized to the REBECCA intervention group. Mean (SD) age was 56 (11) years with only small between-group differences. Patients receiving the REBECCA intervention compared with standard care had reduced (although not significant) symptoms of distress, especially at the 12-month follow-up (estimated effect = −0.51 [95% CI, −1.05 to 0.04]; effect size [ES] = −0.49). Significant effects were seen for symptoms of depression at 6 months (estimated effect = −1.39 [95% CI, −2.33 to −0.44]; ES = −0.27), and breast cancer–specific health-related quality of life at 12 months (estimated effect = 4.03 [95% CI, 1.28- 6.77]; ES = 0.31). Nonsignificant reductions were seen for symptoms of anxiety at 6 months (estimated effect = −1.00 [95% CI, −1.95 to −0.06]; ES = −0.21) and 12 months (estimated effect = −1.01 [95% CI, −1.97 to −0.04]; ES = −0.21), and a nonsignificant increase was seen for patient activation at 18 months (estimated effect = 3.52 [95% CI, −0.09 to 7.12]; ES = 0.25). Stronger intervention effects were observed for younger age, low patient activation, less education, and low social support.

**Conclusions and Relevance:**

Results of this study indicate that patients with breast cancer who were psychologically vulnerable (ie, having moderate to high psychological distress) did not experience significant reduction in distress with nurse navigation. Further research is needed to develop the intervention’s framework and investigate its potential use in clinical practice.

**Trial Registration:**

ClinicalTrials.gov Identifier: NCT03254875

## Key Points

**Question:**

Does nurse navigation alleviate symptoms in patients with psychological vulnerability and breast cancer?

**Findings:**

In this randomized clinical trial of 309 females with breast cancer, nurse navigation did not demonstrate significant reductions in distress.

**Meaning:**

Findings of this trial did not show significant reductions in distress among patients with breast cancer with the nurse navigation intervention. Further research is warranted to develop the nurse navigation framework and explore its potential use in clinical practice.

## Teaser

This randomized clinical trial compares the psychological and physical symptoms among female patients with breast cancer who received a nurse navigation intervention vs usual care over an 18-month period.

## Introduction

With the growing cancer survivor population,^1^ it is increasingly important to find effective ways to address treatment-related symptoms and to target care for patients who are most in need. In patients with breast cancer, prevalent physical and psychological symptoms,^2^ including distress, anxiety and depression,^3^ pain^4,5^ and fatigue,^6^ and distress at diagnosis, may be associated with long-term distress^3^ and physical symptoms such as pain.^5^ Although some patients with breast cancer are able to recover and return to a quality of life corresponding to that of the general population,^7^ a large group of patients report unmet needs for both emotional and physical concerns,^8,9^ which have potential implications for health-related quality of life (HRQOL), treatment adherence,^10^ and prognosis.^11^ Patients with breast cancer who experience high distress at diagnosis may be especially at risk for later physical symptoms.^4^

Nurse navigation^12,13,14,15,16^ and similar approaches provided by specialist breast cancer nurses (SBCNs)^17,18^ focus on collaborative care in which the nurses address both physical and psychological symptoms and refer patients to relevant specialists^19^ by being patient-centered and engaging patients in their own care,^20^ which may be pivotal for patient satisfaction as well as for sustainable symptom reduction. The effects of nurse navigation have been mixed,^12,13,14,15,16^ although SBCN interventions have shown small but consistent improvements in breast cancer–specific HRQOL, anxiety, and depression.^18^ The benefits of these interventions in specific patient subgroups remain largely unexplored. We need to identify and further evaluate promising nurse navigation approaches and to target patients who may benefit the most from them.

The REBECCA (Rehabilitation After Breast Cancer) intervention was developed to target both psychological and physical symptoms through patient-centered and collaborative care, combining for the first time, to our knowledge, nurse navigation and systematic symptom screening in patients with breast cancer who were psychologically vulnerable, defined as having moderate to high psychological distress.^21^ The pilot randomized clinical trial (RCT) showed the promising effects of REBECCA on distress, anxiety, and depression at the 12-month follow-up.^21^ Thus, in this current full-scale RCT, we aimed to examine the long-term effects of the REBECCA nurse navigation intervention compared with usual care in patients with breast cancer and symptoms of distress. We hypothesized that patients receiving the intervention would experience beneficial effects on psychological distress (the primary outcome) and on anxiety, depression, breast cancer–specific HRQOL, fear of recurrence, sleep, cognitive function, health behavior, and need for support (the secondary outcomes) compared with the standard care group.

## Methods

### Study Design and Participants

The study design was a parallel RCT, and the participants were females with breast cancer who were psychologically vulnerable. The trial protocol (Supplement 1) was approved by the Regional Research Ethics Committee. Data sharing was not possible due to European Union General Data Protection Regulation. All participants provided written informed consent. We followed the Consolidated Standards of Reporting Trials (CONSORT) reporting guideline.

Between August 2017 and October 2019, all patients at the Department of Breast Surgery in Rigshospitalet in Copenhagen, Denmark, were recruited and evaluated for eligibility by 3 trained project nurses (H.D. and B.M.). The inclusion criteria were new (prior to treatment) diagnosis of primary breast cancer; breast cancer surgery; aged 18 years or older; Danish speaking; physically able to attend rehabilitation; able to provide written informed consent; and moderate to high psychological distress, with a score of 7 or higher on the well-validated instrument Distress Thermometer (“Please circle the number [0-10] that best describes how much distress you have been experiencing in the last week including today”), as established previously in Danish patients with breast cancer.^22^ Exclusion criteria were severe cognitive problems or dementia and unmanaged psychiatric disease that prevented participation, such as schizophrenia, alcohol use disorder, or narcotic dependence.

Patients were involved in the development of the REBECCA intervention through a previous longitudinal study on symptoms and need for support during breast cancer treatment^3,5,6,8^ and through reporting acceptability in the pilot RCT.^21,23^ Patients expressed high satisfaction, and thus only minor adjustments were made, such as creating electronic rather than paper-based questionnaires.

### Randomization

**Figure 1.**
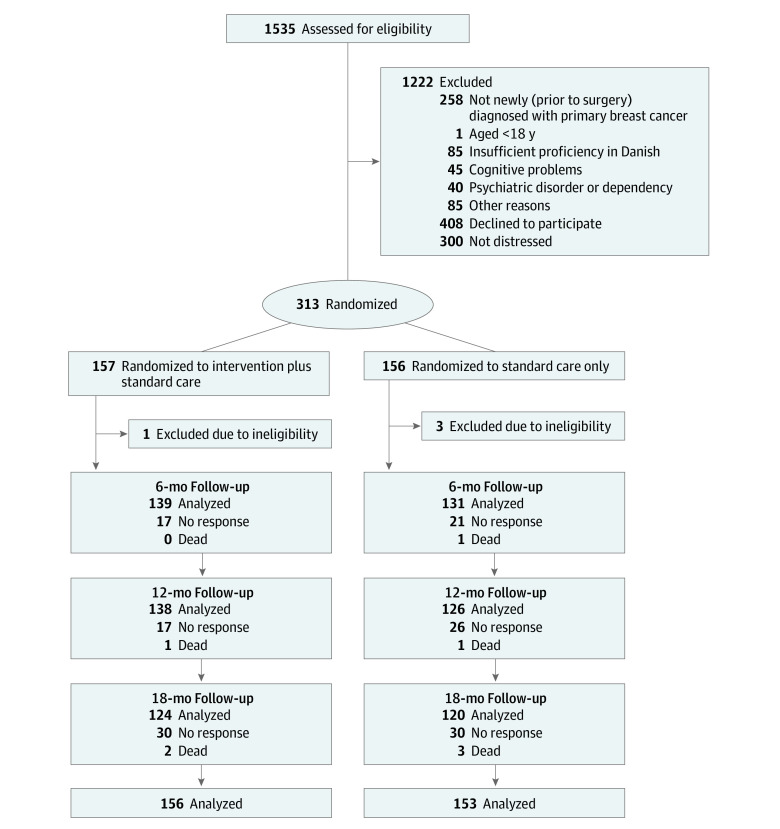
Participant Flow Diagram At baseline, 156 patients in the intervention group and 153 in the standard care group were analyzed.

Enrolled patients were randomized 1:1 by the 3 project nurses using computer-generated assignment and were stratified according to age (<60 or ≥60 years) and treatment modality (none, adjuvant chemotherapy, or neoadjuvant chemotherapy). Patients were randomized to either the standard care or the REBECCA intervention plus standard care (Figure 1). Group allocation was concealed from the navigation nurses until after randomization, but blinding during the trial was not possible due to the behavioral intervention.

### Procedures

REBECCA is a manualized intervention with 2 components^21,24^: (1) systematic screening for patient-reported outcomes of psychological and physical symptoms 3, 9, and 18 weeks after baseline (eFigure 1 in Supplement 2) and (2) nurse navigation, including approximately 6 individual sessions within the first 8 months after baseline per patient need (first session was face-to-face at the breast surgery clinic; other sessions could be by telephone) that are typically either full length (<60 minutes) or short (<10 minutes), to check in on patient status per patient need. The sessions were designed to activate patient engagement; enhance patient attitude, knowledge, and self-efficacy in managing their symptoms; and encourage use of existing rehabilitation services at the hospital or local rehabilitation center. Patient-centered techniques are applied, such as (1) empathetic dialogue and forming of an alliance; (2) joint analysis of the situation, including cognitive behavioral therapy techniques such as cognitive restructuring; (3) assessment and prioritizing of needs for support; (4) psychoeducation; (5) goal setting using SMART (Specific, Measurable, Achievable, Relevant, and Time-bound) goals, including (when relevant) referral for management of pain at the oncology department or for management of clinical depression in up to 6 individual sessions with a project psychologist; (6) agreements, homework, and planning; and (7) debriefing.

The REBECCA intervention was delivered by 3 experienced nurses (all of whom had ≥20 years’ experience with different patient groups) who were trained in the manualized sessions through a 3-day program. Patients in both intervention and standard care groups had access to usual care, which included regular treatment and nurse support at chemotherapy and radiotherapy appointments as well as municipality-based rehabilitation, including patient support groups and physical training.

### Outcomes

Questionnaire data were collected between August 2017 and March 2021 either electronically or on paper (based on patient preference) at baseline and at 6, 12, and 18 months after diagnosis. The primary outcome was psychological distress, as measured using the Distress Thermometer^22^ with a score range of 0 to 10 points and a higher score indicating greater distress. The secondary outcomes included (1) symptoms of anxiety measured by the 7-item Generalized Anxiety Disorder,^25^ with a score range of 0 to 21 points and a higher score indicating greater anxiety and a minimally important difference (MID) of 3 points; (2) symptoms of depression measured by the 9-item Patient Health Questionnaire,^26^ with a score range of 0 to 27 points and a higher score indicating higher level of depression and an MID of 5 points; (3) breast cancer–specific HRQOL measured by the Trial Outcome Index–Physical/Functional/Breast score from the Functional Assessment of Cancer Therapy–Breast scale,^27^ with a score range of 0 to 148 points and a higher score indicating greater HRQOL, and an MID of 5 to 6 points; (4) fear of recurrence measured by the 4-item Concerns About Recurrence Questionnaire,^28^ with a score range of 0 to 40 points and a higher score indicating greater fear of recurrence; (5) sleep measured by the Pittsburgh Sleep Quality Index,^29^ with a score range of 0 to 21 points and a higher score indicating worse sleep; (6) cognitive function and perceived cognitive impairment measured using the Functional Assessment of Cancer Therapy–Cognitive Function^30^ scale, with a score range of 0 to 72 points and a higher score indicating better function; (7) patient activation as an indicator of self-efficacy measured by the Patient Activation Measure,^31^ with a score range of 0 to 100 points and a higher score (>67 points) indicating greater activation; (8) pain measured using the Neuropathic Pain Scale for Postsurgical Patients,^32^ with a score range of 0 to 5; (9) health behavior, such as smoking, alcohol use, and physical activity; (10) body mass index, which was calculated as weight in kilograms divided by height in meters squared; and (11) need for support.

Baseline data were collected on demographic characteristics (age: <60 or ≥60 years^33^); years of education (<10, 10-12, >12-15, or >15 years); employment status (not employed or employed); cohabitating partner (no or yes); and social support measured with the short version of the Medical Outcomes Social Support Survey,^34^ with scores in the higher quantile (≥25%) indicating better support (eFigure 1 in Supplement 2). Because we were not able to include patients who were not Danish speaking, no information was collected on ethnicity.

From medical records, we obtained information on breast surgery (lumpectomy, mastectomy, or mastectomy with primary reconstruction), axillary surgery (axillary dissection or sentinel node biopsy), adjuvant radiotherapy (no or yes), adjuvant endocrine therapy (no or yes), chemotherapy (adjuvant, neoadjuvant, or none), and trastuzumab (no or yes). Additionally, nurse registrations on intervention exposure (3 single items) and patient-reported acceptability (7 single items) were obtained in the intervention group.

### Power Considerations

An a priori sample size evaluation was established based on results regarding distress (primary outcome) as well as symptoms of depression and anxiety (secondary outcomes) from the pilot RCT, in which up to 30% between-group difference was observed in change from case to noncase in distress, anxiety, and depression.^21^ In this full-scale trial, we expected a conservative 20% between-group difference. With a 2-year recruitment, we expected to recruit 324 patients, with approximately 20% having missing data or dropping out, resulting in a total of 260 participants (130 in each group) and a power of 84%.

### Statistical Analyses

Deviations from the original analysis plan were made and finalized prior to data analyses and are described here. Descriptive analyses were applied to examine the differences between study groups at baseline. In intention-to-treat analyses, we applied linear mixed-regression models to examine the effect of the intervention on the primary outcome (distress) and secondary continuous outcomes (symptoms of anxiety and depression, breast cancer–specific HRQOL, fear of recurrence [no baseline data were included, and thus different effects of the intervention were allowed at all time points], sleep, cognitive function, and patient activation) in repeated-measures analyses, with a random effect for patients at 4 time points: baseline and 6-, 12-, and 18-month follow-up. The models assumed no difference between groups at baseline and were in the revised analysis plan, adjusted for randomization strata of age (<60 or ≥60 years) and treatment modality (none, adjuvant chemotherapy, or neoadjuvant chemotherapy).

Statistical significance was interpreted at 2-sided *P* < .01 to accommodate multiple testing. However, in the revised analysis plan, 99% CIs were replaced by 95% CIs for comparability with other studies. Effect size was evaluated using Cohen *d*. In the revised analysis plan, we included visual comparisons of the strength of the associations between the outcomes in forest plots, where we fitted models with standardized outcome scores by subtracting the sample mean score from each score and dividing by the SD across time points. Logistic regression models were added to the revised analysis plan to examine the differences between study groups at 18 months in unmet need for support and health behavior, adjusted for baseline outcome values.

In the revised analysis plan, we added exploratory analyses to examine whether the intervention effects were modified by age, years of education, social support, patient activation, and chemotherapy, using an interaction term between treatment group and the specific effect modifier. We conducted sensitivity analyses to examine the potential effect of missing data. The last-observation-carried-forward method in the revised analysis plan was replaced by multiple imputations using fully conditional specification methods^35^ to impute missing values for the outcomes at different follow-up times, with information from covariates (years of education, breast surgery, axillary surgery, adjuvant radiotherapy, adjuvant endocrine therapy, and chemotherapy) and with values from the same patient imputed together. Two scenarios were examined: assuming that data were missing at random, and assuming patients with missing data had 20% worse symptoms than expected.

Intervention exposure and acceptability were assessed descriptively. Analyses were performed using R, version 4.0.4 (R Core Team)^36^ from June 2021 to October 2022.

## Results

A total of 1535 patients were evaluated for eligibility, of which 1222 were excluded (514 were not eligible, 408 declined, and 300 were not distressed). After exclusions, 313 patients were randomized to either the intervention (n = 157) or standard care (n = 156) group (Figure 1). However, 4 patients were withdrawn after randomization as they had not received surgery and did not fulfill inclusion criteria. Thus, 309 female patients were included for analyses in the intervention (n = 156) and standard care (n = 153) groups. These patients had a mean (SD) age of 56 (11) years, included 207 (67%) living with a partner, and varied little across intervention and standard care groups (Table 1). During follow-up, 8 patients (3 in the intervention group, and 5 in the standard care group) died, no patients dropped out, and all attrition was due to nonresponse to questionnaires (Figure 1). Participants in the intervention group and the standard care group, had high levels of distress (mean [SD] score, 8.20 [1.09] vs 8.22 [1.03]), anxiety (mean [SD] score, 10.67 [5.21] vs 10.96 [4.88]), and depression (mean [SD] score, 7.84 [5.05] vs 8.34 [5.13]) at baseline, which declined over time (Table 2).

**Table 1.** Baseline Characteristics of Randomized Patients by Study Group

Variable	Study group, No. (%)	
	Intervention	Standard care
All patients, No.	156	153
Age at baseline, mean (SD), y	55.8 (11.1)	56.7 (11.4)
Cohabitating partner		
No	53 (34)	49 (32)
Yes	103 (66)	104 (68)
Years of education^a^		
<10	12 (8)	5 (3)
10-12	19 (12)	28 (18)
>12-15	98 (63)	95 (62)
>15	26 (17)	25 (16)
Employment status		
Not employed^b^	11 (7)	13 (9)
Employed	112 (72)	100 (65)
Receiving age-related pension	33 (21)	40 (26)
Breast surgery		
Lumpectomy	80 (51)	96 (63)
Mastectomy	67 (43)	52 (34)
Mastectomy with primary reconstruction	9 (6)	5 (3)
Axillary surgery		
Axillary dissection	31 (20)	26 (17)
Sentinel node biopsy	125 (80)	127 (83)
Adjuvant radiotherapy		
No	51 (33)	37 (24)
Yes	105 (67)	116 (76)
Chemotherapy		
Adjuvant	73 (47)	68 (44)
Neoadjuvant	31 (20)	28 (18)
None	52 (33)	57 (37)
Adjuvant endocrine therapy		
No	37 (24)	29 (19)
Yes	119 (76)	124 (81)
Trastuzumab		
No	136 (87)	128 (84)
Yes	20 (13)	25 (16)

^a^ One person had missing data on years of education.

^b^ Not employed included women with less education, unemployed status, other government support than age-related pension, and missing information.

**Table 2.** Primary and Secondary Outcomes at Follow-up

Intervention effect	Mean score (SD)		Linear mixed-regression model^a^		
	Intervention (n = 156)	Standard care (n = 153)	Estimated effect (95% CI)	*P* value	Effect size, Cohen *d*
Distress^b^					
Baseline	8.20 (1.09)	8.22 (1.03)	NA	NA	NA
6-mo Follow-up	3.64 (2.66)	4.15 (2.61)	−0.46 (−1.00 to 0.08)	.09	−0.45
12-mo Follow-up	3.18 (2.73)	3.67 (2.98)	−0.51 (−1.05 to 0.04)	.07	−0.49
18-mo Follow-up	2.98 (2.90)	3.01 (2.80)	−0.03 (−0.60 to 0.53)	.91	−0.03
Overall effect	NA	NA	NA	.15	
Anxiety^b^					
Baseline	10.67 (5.21)	10.96 (4.88)	NA	NA	NA
6-mo Follow-up	3.74 (4.03)	4.85 (4.72)	−1.00 (−1.95 to −0.06)	.04	−0.21
12-mo Follow-up	3.27 (4.08)	4.31 (4.22)	−1.01 (−1.97 to −0.04)	.04	−0.21
18-mo Follow-up	3.44 (4.12)	4.55 (4.90)	−0.94 (−1.93 to 0.05)	.06	−0.19
Overall effect				.06	
Depression^b^					
Baseline	7.84 (5.05)	8.34 (5.13)	NA	NA	NA
6-mo Follow-up	4.42 (4.08)	6.05 (4.80)	−1.39 (−2.33 to −0.44)	.004	−0.27
12-mo Follow-up	4.12 (4.34)	4.82 (4.39)	−0.62 (−1.57 to 0.33)	.20	−0.12
18-mo Follow-up	4.17 (4.41)	4.75 (4.28)	−0.33 (−1.31 to 0.66)	.52	−0.06
Overall effect	NA	NA	NA	.04	
HRQOL^c^					
Baseline	71.82 (12.52)	71.79 (12.96)	NA	NA	NA
6-mo Follow-up	68.95 (15.02)	66.36 (15.94)	2.52 (−0.20 to 5.23)	.07	0.19
12-mo Follow-up	73.37 (14.03)	69.56 (15.44)	4.03 (1.28 to 6.77)	.004	0.31
18-mo Follow-up	73.13 (14.63)	70.67 (15.52)	2.32 (−0.51 to 5.15)	.11	0.18
Overall effect	NA	NA	NA	.03	
Sleep function^b^					
Baseline	7.17 (3.96)	7.36 (3.89)	NA	NA	NA
6-mo Follow-up	7.34 (3.75)	7.77 (4.24)	−0.25 (−1.02 to 0.53)	.53	−0.06
12-mo Follow-up	7.20 (4.15)	7.12 (4.13)	0.11 (−0.67 to 0.89)	.78	0.03
18-mo Follow-up	6.80 (4.09)	7.01 (3.91)	0.04 (−0.76 to 0.84)	.92	0.01
Overall effect	NA	NA	NA	.86	
Fear of recurrence^b^^,^^d^					
6-mo Follow-up	12.81 (9.77)	14.62 (10.70)	−2.06 (−4.51 to 0.39)	.10	−0.2^e^
12-mo Follow-up	12.76 (10.29)	14.75 (10.27)	−2.09 (−4.53 to 0.35)	.09	−0.2^e^
18-mo Follow-up	12.38 (10.03)	13.88 (10.07)	−1.40 (−3.9 to 1.1)	.27	−0.14^e^
Overall effect	NA	NA	NA	.32	
Patient activation^c^					
Baseline	61.75 (17.26)	61.51 (14.14)	NA	NA	NA
6-mo Follow-up	63.34 (15.64)	62.81 (14.36)	1.39 (−2.07 to 4.85)	.43	0.1
12-mo Follow-up	66.23 (15.59)	63.36 (17.06)	2.97 (−0.5 to 6.44)	.09	0.21
18-mo Follow-up	64.92 (14.50)	61.93 (16.52)	3.52 (−0.09 to 7.12)	.06	0.25
Overall effect	NA	NA	NA	.19	
Cognitive function^c^					
Baseline	57.01 (15.73)	57.24 (13.87)	NA	NA	NA
6-mo Follow-up	55.85 (14.46)	53.89 (15.46)	2.32 (−0.4 to 5.03)	.09	0.17
12-mo Follow-up	55.45 (16.82)	55.00 (14.69)	1.36 (−1.38 to 4.1)	.33	0.1
18-mo Follow-up	57.07 (15.38)	55.37 (15.38)	1.33 (−1.5 to 4.15)	.36	0.1
Overall effect	NA	NA	NA	.40	

Abbreviations: HRQOL, health-related quality of life; NA, not applicable.

^a^ Assuming no difference between groups at baseline, the model was adjusted for randomization strata of age (<60 or ≥60 years) and treatment modality (none, adjuvant chemotherapy, or neoadjuvant chemotherapy).

^b^ Higher score indicating higher symptoms.

^c^ Higher score indicating better quality of life.

^d^ No baseline level to take into account.

^e^ Cohen *d* for fear of recurrence was calculated by dividing the 6-month SD of both groups.

### Intervention Effects

**Figure 2.**
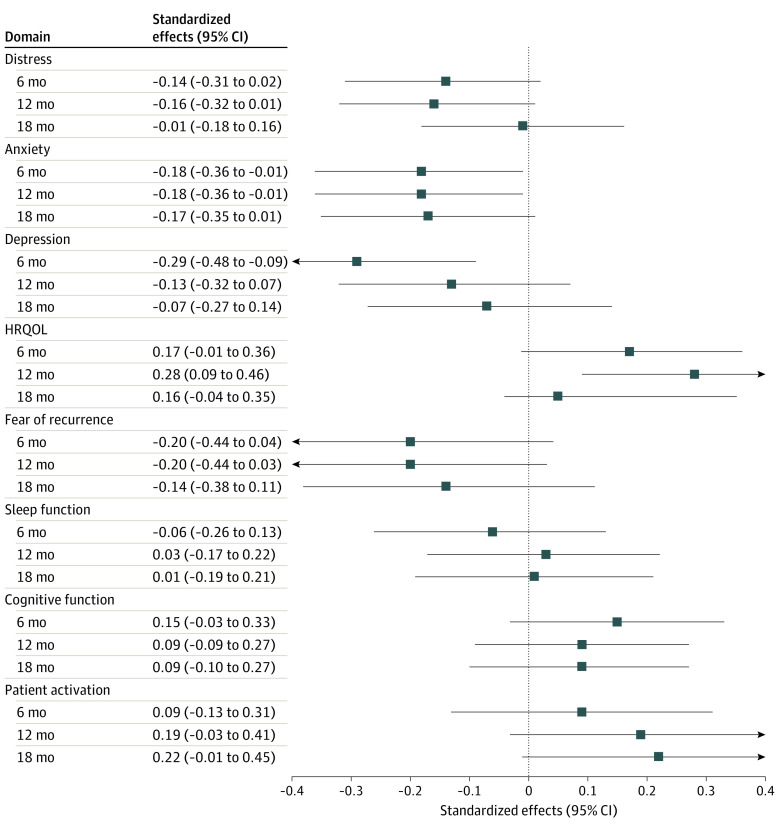
Forest Plot of Standardized Intervention Effects at 6-, 12-, and 18-Month Follow-up in 309 Patients With Breast Cancer Standardized outcome scores were applied using fitted models by subtracting the sample mean score from each score and dividing by the SD. Models were adjusted for randomization strata of age (<60 years or ≥60 years) and treatment modality (none, adjuvant chemotherapy, or neoadjuvant chemotherapy). HRQOL indicates health-related quality of life.

Patients in the intervention group reported lower distress, although not significantly lower, at the 6-, 12-, and 18-month follow-up, with the largest reductions observed at 12 months (estimated effect = −0.51 [95% CI, −1.05 to 0.04]; effect size [ES] = −0.49) (Table 2, Figure 2). Significant reductions were seen for symptoms of depression at 6 months (estimated effect = −1.39 [95% CI, −2.33 to −0.44]; ES = −0.27) and breast cancer–specific HRQOL at 12 months (estimated effect = 4.03 [95% CI, 1.28- 6.77]; ES = 0.31). Changes that were not significant were seen for symptoms of anxiety at 6 months (estimated effect = −1.00 [95% CI, −1.95 to −0.06]; ES = −0.21) and 12 months (estimated effect = −1.01 [95% CI, −1.97 to −0.04]; ES = −0.21) and for patient activation at 18 months (estimated effect = 3.52 [95% CI, −0.09 to 7.12]; ES = 0.25) as well as for fear of recurrence, sleep, and cognitive function (Table 2, Figure 2) or unmet needs for support and health behavior (eTable 1 in Supplement 2). In analyses using imputation models, the intervention effect was similar or stronger in both multiple imputed scenarios (eTable 2 in Supplement 2).

### Subgroup Effects, Exposure, and Acceptability

**Figure 3.**
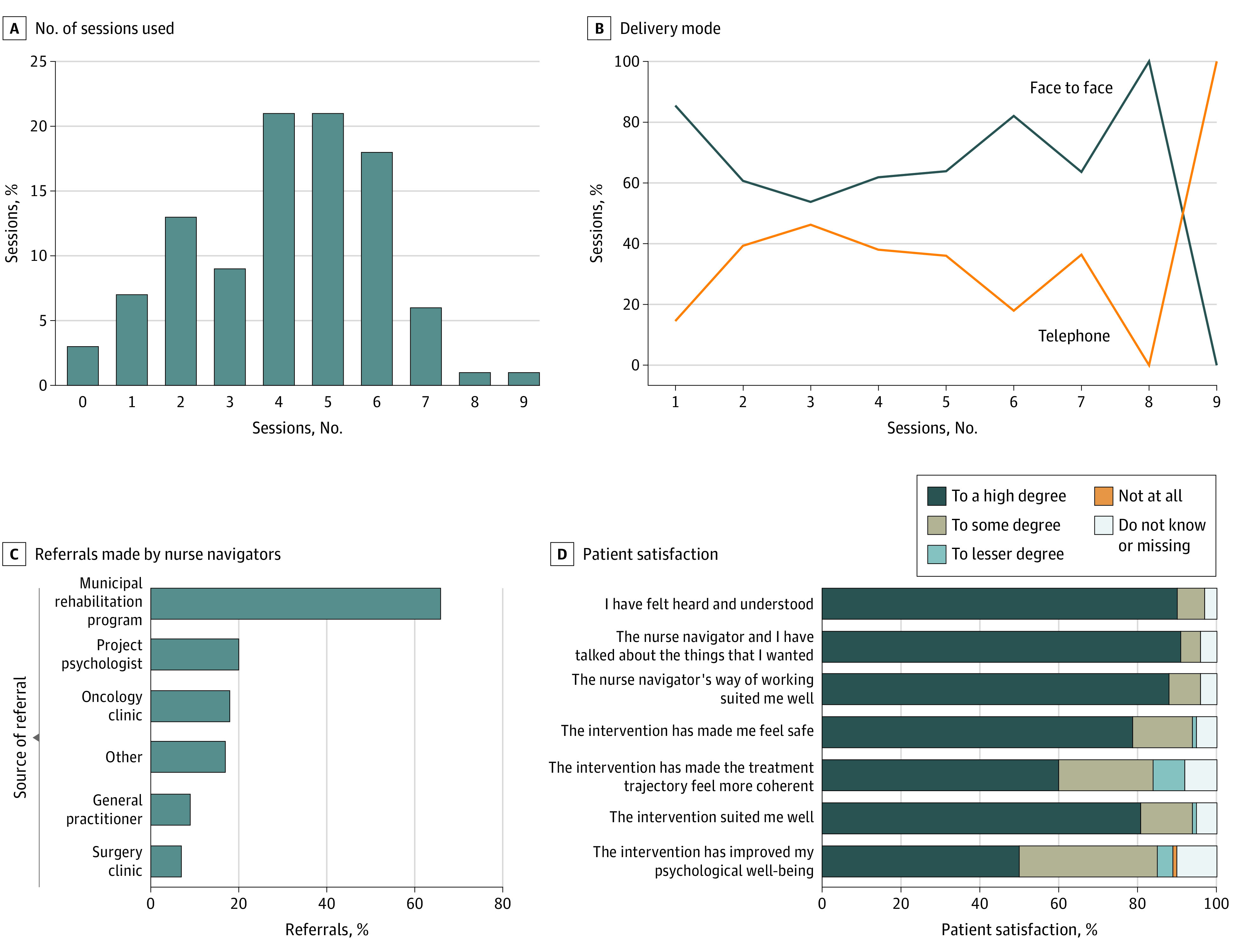
Intervention Delivery Among 156 Patients

Stronger intervention effects were seen for vulnerable subgroups, such as patients 60 years or older, patients with less education, patients with low patient activation, and patients with low social support, with different strengths for the individual outcomes (eFigures 2 to 9 in Supplement 2). For example, in patients with low patient activation, significant effects were seen for distress at the 6-month (estimated effect = −1.35; 95% CI, −2.21 to −0.49) and 12-month (estimated effect = −1.08; 95% CI, −1.95 to −0.22) follow-up. Patients in the intervention group had a median (range) of 4 (0-9) sessions, with most of the sessions being face-to-face and the largest proportion of referrals being municipality-based rehabilitation (42%) (Figure 3A-D). Patient satisfaction was high: 91% of patients discussed the issues most important to them. No harmful effects were reported during the study.

## Discussion

Nurse navigation and systematic screening for symptoms provided in the REBECCA intervention showed promise in reducing several psychological symptoms and increasing quality of life. Reduced, but not significant, intervention effects were observed for the primary outcome of distress, and significant effects were observed for symptoms of depression and breast cancer–specific HRQOL. Additionally, nonsignificant effects were found for symptoms of anxiety and patient activation. The intervention effects did not meet the established MIDs, and small to moderate effect sizes were observed (ES = 0.21-0.31). In the pilot RCT (n = 50), significant improvements in distress were found after 12 months. That the effect on distress was not significant has no obvious explanation, but a possible reason is that the effect was especially pronounced in patients with few resources, as we found significant effects at all follow-up periods for patients with fewer than 12 years of education and for patients with low patient activation.

### Nurse Navigation

Previous nurse navigation intervention studies rarely demonstrated substantial symptom reduction, such as studies on patients with breast cancer or mixed cancer (including breast) that found no significant effects of nurse navigation on distress, fatigue, quality of life, and health care use (n = 251)^13^; studies on breast cancer–related quality of life (n = 251)^16^; or studies on patient experience outcomes according to depression (n = 190).^12^ Still, 6 RCTs on SBCN interventions showed small improvements in cancer-specific HRQOL as well as anxiety and depression.^18^

The main strength of this RCT is that, to our knowledge, it was the first to test the effect of a manualized nurse navigation program on female patients with high distress levels at diagnosis, thus minimizing a floor effect, and it was also the first to examine long-term effects over 18 months. Previous nurse navigation interventions were only superficially described and covered a variety of techniques, including addressing needs,^12,13,14^ referral to services (eg, for depression),^12,13,14^ counseling,^12,13,14^ and inclusion of an informal caregiver if relevant,^13^ whereas SBCN interventions during treatment were often based on minimally described counseling.^18^ In further development of nurse navigation, rigorousness should be applied to the theoretical intervention framework and specific techniques should be applied. The high participation rate of 60% indicates that the intervention was relevant to a broad range of patients with breast cancer, which supports the generalizability of the study results.

The REBECCA intervention may work through several pathways: systematic screening for patient-reported outcome symptoms may enhance the detection and professional management of the symptoms.^37^ Concurrently, through the patient-centered approach, the nurse navigation may enhance more needs-based support as well as patient motivation and skills in self-managing the symptoms. For several outcomes, the intervention had a stronger effect on patients with limited social support, low patient activation, a lower level of educational attainment, and age 60 years or older, suggesting that the intervention effects may be strongest in patients with social vulnerabilities. To our knowledge, supportive care interventions have rarely examined the differences in effectiveness across patients with different levels of resources. Some studies have suggested that it may be feasible to address socioeconomic inequality in cancer care through supportive care interventions, yet the evidence on their effects on symptoms is still limited.^38^ Due to the challenges of inequality in cancer care, it is essential to further explore if the REBECCA intervention and similar supportive care interventions may have the greatest benefits for cancer populations with psychological symptoms and/or social vulnerabilities. The REBECCA intervention is delivered through a median of 4 nurse sessions, has a mixed telephone and physical format, and is potentially cost-effective; however, we plan to investigate its cost-effectiveness in a separate study.

### Limitations

This study has limitations. With 120 patients per group included at the 18-month follow-up (Figure 1), the study did not completely achieve the recruitment goal of 130 per group, slightly limiting the power of the study. However, with this large study size, we expected the effect to be minimal. Due to the number of analyses conducted, we applied a strict significance level (*P* < .01), but the results of the secondary interaction analyses should still be considered as exploratory and should be interpreted with caution. As anticipated, over an 18-month follow-up period, we observed attrition of up to 22%; however, similar attrition rates were seen across groups and sensitivity analyses. Applying different attrition scenarios supported the strong intervention effects. We did not obtain information on ethnicity, and as eligibility required understanding and speaking Danish, we cannot exclude the limited generalizability to ethnic minority groups.

## Conclusions

We believe the REBECCA intervention fills an important gap in the existing literature regarding providing patient-centered care to patients with breast cancer and social and psychological vulnerabilities. In this RCT, we observed reduced distress in patients who received the REBECCA intervention, especially after 12 months, although the effect was not significant. The intervention resulted in significant improvement in symptoms of depression and breast cancer–specific HRQOL, especially at the 6- and 12-month follow-up, and nonsignificant improvement in symptoms of anxiety and patient activation. The effect sizes were small, but effects were especially pronounced in subgroups with social vulnerabilities, and patient satisfaction was high. To our knowledge, this is the first trial to show the feasibility (through a simple triage approach) of systematically selecting patients with breast cancer who had psychological symptoms of distress and to offer them more comprehensive supportive care, with the nurse navigator actively supporting the patient in accessing health care services that are available within the health care system. These findings warrant further research to develop the nurse navigation framework and to explore the potential translation of this intervention into clinical practice.

## Notes

Supplement 1.
Trial Protocol

Supplement 2. **eTable 1.** Need for Support and Health Behaviour Outcomes at 18 Months Follow-up in Patients With Breast Cancer in the REBECCA Study **eTable 2.** Primary and Secondary Outcomes Using Multiple Imputations at Random and Not at Random at 6-, 12-, and 18-Months Follow-up in 309 Patients With Breast Cancer in the REBECCA Study **eFigure 1.** Schedule of Enrollment, Interventions and Assessment in the REBECCA Randomized Controlled Trial **eFigure 2.** Standardized Interaction Effects for Distress at 6, 12, and 18 Months Follow-up in 309 Patients With Breast Cancer in the REBECCA Study **eFigure 3.** Standardized Interaction Effects for Anxiety at 6, 12, and 18 Months Follow-up in 309 Patients With Breast Cancer in the REBECCA Study **eFigure 4.** Standardized Interaction Effects for Depression at 6, 12, and 18 Months Follow-up in 309 Patients With Breast Cancer in the REBECCA Study **eFigure 5.** Standardized Interaction Effects for Breast Cancer-Specific HQoL at 6, 12, and 18 Months Follow-up in 309 Patients With Breast Cancer in the REBECCA Study **eFigure 6.** Standardized Interaction Effects for Sleep Function at 6, 12, and 18 Months Follow-up in 309 Patients With Breast Cancer in the REBECCA Study **eFigure 7.** Standardized Interaction Effects for Fear of Recurrence at 6, 12, and 18 Months Follow-up in 309 Patients With Breast Cancer in the REBECCA Study **eFigure 8.** Standardized Interaction Effects for Patient Activation at 6, 12, and 18 Months Follow-up in 309 Patients With Breast Cancer in the REBECCA Study **eFigure 9.** Standardized Interaction Effects for Cognitive Function at 6, 12, and 18 Months Follow-up in 309 Patients With Breast Cancer in the REBECCA Study

Supplement 3.
Data Sharing Statement
