# Supplementary material for: Effect of a Nurse Navigation Intervention on Mental Symptoms in Patients With Psychological Vulnerability and Breast Cancer: The REBECCA Randomized Clinical Trial
Source: JAMA Netw Open. 2023 Jun 23;6(6):e2319591. doi: 10.1001/jamanetworkopen.2023.19591 (PMC10290249; doi:10.1001/jamanetworkopen.2023.19591)
Supplement: Supplement 1. — Trial Protocol [file jamanetwopen-e2319591-s001.pdf]

Protokol
Appendiks 2. Protokol_REBECCA_v2_060417
Titel: REBECCA Forskning i rehabilitering efter brystcancer
Title: REBECCA: Individually tailored nurse navigation for distressed breast cancer patients
This protocol was submitted and approved by the Danish Ethics Committee in 2017.

Protocol by

Pernille Bidstrup, senior researcher, psychologist, PhD[1]
Birgitte Goldschmidt Mertz, RN [2]
Niels Kroman, professor, MD, DMSc[2]
Christoffer Johansen, professor, MD, MDSc[1,3]

Danish Cancer Society Research Center[1]
Section of Breast Surgery, Rigshospitalet[2]
Oncology Clinic, Rigshospitalet[3]

Further, the study advisory board includes:

- Oncology Clinic, Rigshospitalet
    - Michael Andersson, Ulla Breitenstein Mathiesen
- Oncology Clinic, Herlev Hospital
    - Gosia Tuxen
- Copenhagen Centre for Cancer and Health
    - Jette Vibe-Petersen
- The Danish Cancer Society Research Center
    - Susanne Dalton
- Section for surgical pathophysiology, Rigshospitalet
    - Kenneth Geving Andersen

CONTENT

**Synopsis**	**3**
**Background**	**4**
**Aim**	**4**
**Material and methods**	**5**
**Design**	**5**
**Participants**	**5**
**Inclusion**	**5**
**Randomization**	**6**
**Intervention**	**7**
**Data collection**	**8**
**Questionnaires**	**8**
**Registries and clinical databases**	**9**
**Outcomes**	**9**
**Statistical analyses**	**10**
**Power and participation**	**10**
**Organization**	**11**
**Ethics**	**11**
**Dissemination**	**12**
**Perspectives**	**13**
**Funding**	**13**
**References**	**14**

SYNOPSIS

**Title**	REBECCA: Individually tailored nurse navigation for distressed breast cancer patients
**Objectives**	to evaluate whether a screening-based individually tailored nurse navigator intervention compared to standard care significantly reduce symptoms among women being treated for breast cancer who had moderate-to-severe distress (score ≥ 7 on the distress thermometer).
**Design**	Two armed RCT
**Outcomes**	Primary outcome: psychological distress in the intervention group compared to the control group.<br>Secondary outcomes: improvement in anxiety, depression, fear of recurrence, breast cancer specific symptoms, quality of life, patient activation, self-management, work ability, total health care utilization, financial costs
**Inclusion**	Criteria include: Newly diagnosed with breast cancer (i.e. prior to surgery), scheduled for breast cancer surgery at Section of Breast Surgery at Rigshospitalet, 18 years or above, Danish citizen, read, understand and speak Danish, female gender, no severe cognitive problems or dementia, no severe psychiatric disease requiring treatment, e.g. schizophrenia, alcohol or narcotic dependence, has signed written informed consent. Those who experience high psychological distress (score ≥ 7 on the distress thermometer) will enter the RCT whereas those who experience low psychological distress (score < 7 on the distress thermometer) will be followed in an observational questionnaire study.
**Method**	During a 2-year period, 758 patients newly diagnosed with breast cancer will be recruited from the Section of Breast Surgery at Rigshospitalet, Copenhagen. Of these 324 participants enter the RCT and are randomized in a computer-generated sequence of 1:1 to the intervention (nurse-navigation) or control group (standard care). A total of 432 participants will enter an observational questionnaire study. All participants are followed for 1,5 years.
**Intervention**	The intervention is a program of individually tailored nurse navigation for distressed breast cancer patients. The specific change objectives are to improve knowledge, social norms and self-efficacy so that the patient can self-manage or actively engage in professional rehabilitation to alleviate her psychological and physical symptoms. The intervention consists of systematic report of symptoms to the nurse and up to six individual, manual-based, face-to-face or telephone nurse navigator sessions depending on individual needs.
**Control**	Patients in the control group will receive standard rehabilitation and care
**Data collection**	We will collect data using electronic questionnaires, clinical databases, and national registers before intervention.
**Investigators/center**	The Danish Cancer Society Research Center, Section of Breast Surgery, Rigshospitalet and Oncology Clinics at Rigshospitalet and Herlev Hospital.

BACKGROUND
The current study is a full-scale version of a pilot study, which was approved by De Videnskabsetiske Komiteer for Region Hovedstaden (J.nr. H-1-2013-030).

Women with breast cancer experience a number of psychological and physical symptoms during treatment (1), which are not always adequately managed. Although some symptoms attenuate over time, certain patients may have continuously severe symptoms even after the end of treatment (2). In Denmark, a municipal rehabilitation system is used to help manage such symptoms, in collaboration with hospitals and general practitioners. Nevertheless, cancer patients, including women being treated for breast cancer, still report that they need support and symptom management (3). The greatest challenges in cancer care reported by patients, caregivers and care providers are: delay and lack of coordination of care, lack of information and inadequate management of psychological and social problems (4).

Many recent attempts to optimize and integrate cancer rehabilitation have involved one of two overall approaches: screening for symptoms on the basis of patient-reported outcome measurements (PROMs) and nurse navigation. Using PROMs may improve communication between patients and health professionals (5), but the effect on symptoms is not clear. In a review of studies on improving patient outcomes by using PROMs, Kotronoulas (2014) identified 24 controlled trials with wide variation in the content of the interventions delivered (6). The authors concluded that using PROMs had only a small-to-moderate effect on patient outcomes such as physical and psychological symptoms. To our knowledge, only three randomized controlled trials (RCTs) have been conducted on oncology navigation programs (7-9) and none of them were based only on patients with psychological distress.

Study rational
Although the studies suggest that nurse navigation results in a positive patient experience and fewer problems in care, none of the studies indicates an effect of navigation on patient-reported psychological or physical symptoms. Thus, although it is recognized that cancer patients have treatment-related symptoms, evidence-based models tested in high-quality RCTs are required for optimal management of cancer-related symptoms.

We have developed an individually tailored nurse navigator intervention (10) and have performed a randomized pilot test with 50 women with breast cancer. The study was approved by De Videnskabsetiske Komiteer for Region Hovedstaden (J.nr. H-1-2013-030). The aim of the pilot study was to test the feasibility and effect of a screening-based individually tailored nurse navigator intervention entitled REBECCA for women being treated for breast cancer who had moderate-to-severe distress (score ≥ 7 on the distress thermometer) in a randomized controlled design. The results, which we have submitted for publication in a scientific journal, show very promising feasibility as well as statistically significant effects of an individually tailored nurse-navigation intervention on the psychological symptoms distress and anxiety. The breast cancer patients as well as the breast cancer health care professionals have provided positive feedback and the health care system requests alternative approaches to risk-stratified care and support. We have thus now established the scientific and clinical basis to continue the development of the intervention and test it in a full-scale study.

AIM

The aim of the randomized controlled trial is to evaluate whether a screening-based individually tailored nurse navigator intervention entitled Rebecca compared to standard care significantly reduce symptoms among women being treated for breast cancer who had moderate-to-severe distress (score ≥ 7 on the distress thermometer).

MATERIAL AND METHODS

Design

The effect of the REBECCA intervention will be evaluated in a two-armed randomized controlled trial testing whether a screening-based individually tailored nurse navigator intervention entitled Rebecca compared to standard care significantly reduce symptoms among women being treated for breast cancer who had moderate-to-severe distress (score ≥ 7 on the distress thermometer).

Participants

During a 2-year period, the patients will be recruited from the Section of Breast Surgery at Rigshospitalet, Copenhagen and we expect to include 758 patients in the study.

To be included, patients have to fulfill the following criteria:

- Newly diagnosed with breast cancer (i.e. prior to surgery)
- Scheduled for breast surgery at Section of Breast Surgery at Rigshospitalet
- Age 18 or above
- Danish citizen
- Read, understand and speak Danish
- Female gender
- No severe cognitive problems or dementia
- No severe psychiatric disease requiring treatment, e.g. schizophrenia, alcohol or narcotic dependence
- Has signed written informed consent

Those who experience high psychological distress (score ≥ 7 on the distress thermometer) will enter the RCT (N=324) whereas those who experience low psychological distress (score < 7 on the distress thermometer) will be followed in an observational questionnaire study.

The patients who receive surgery at the Section of Breast Surgery at Rigshospitalet will later receive oncological treatment at the oncology clinics at either Rigshospitalet or Herlev Hospital and rehabilitation at the local rehabilitation center of their municipality.

Inclusion procedure

Newly diagnosed consecutive breast cancer patients will be pre-screened for eligibility and invited to participate at the Section of Breast Surgery at Rigshospitalet, Copenhagen, Denmark, between April 2017 and April 2019.

On the day the operation is planned (average 14 days prior to surgery), eligible patients will receive a short introduction and written information (Appendix 3) about the study including leaflet published by the National Ethical Committee “Before you make up your mind” (Før du beslutter dig) (Appendix 4), in the outpatient clinic. The patient will be informed, that there

will be talked about this randomized controlled trial at the next visit and that she is welcome to bring a relative to the consultation.

At the last pre-surgery visit (within few days of surgery and thus after a consideration time of minimum 24 hours), one of the three project navigator nurses will provide further verbal information about the REBECCA study, give the patient the opportunity to ask more questions and will hand out an informed consent form along with the a questionnaire. The nurse will follow the guidelines for obtaining informed consent as described (Appendix 5). Patients who attend alone and would like to bring a relative before deciding, will be offered an additional consultation with the presence of a relative.

Patients who agree to participate in the study are asked to sign the informed consent forms (Appendix 6, 6A) and return it along with the questionnaire. When the nurse has received the signed informed consent form and the questionnaire, she will access a computer program and perform the randomization. The nurse will e-mail or post a printed copy of the informed consent form to all participants.

Randomization
Women who are distressed (score ≥ 7 on the distress thermometer) (N=324) will be included in the randomized controlled trial and randomized in a computer-generated sequence of 1:1 to the intervention or control group, with stratification by age (< 60 and > 60 years), generating a balanced number of random assignments to the two groups in blocks of randomly varying sizes of six, eight or ten patients securing an equal distribution of patients in both groups. The randomization is blinded. Thus, neither the investigators nor the patients can influence the group to which each patient is allocated. An electronic platform will be developed by an experienced IT consultant company, to register, randomize and track included patients. The same platform will be used for the intervention activities and data collection.

Immediately after randomization, the nurse will inform patients about group allocation and consequences for further study participation. The nurse will schedule the first appointment with the patients in the intervention group.

Women who are not distressed (score < 7) (N=432), will participate in an observational group following the same procedure as the control group.

Intervention
The intervention includes two major components:

- **(i)** An electronic platform to report symptoms to the nurses
- **(ii)** Nurse navigation focusing on symptom evaluation and recognition, as well as management of these symptoms through self-management or referral

Conceptual model
The development of the intervention program has been described in the publication by Bidstrup et al. (10). The aim of the intervention is to improve patient-reported psychological and physical symptoms. By applying the social-cognitive “stages of change” model, a lowered symptom burden will be sought by creating individual behaviour change (11). The specific change objectives are to improve knowledge, social norms and self-efficacy so that

the patient can self-manage or actively engage in professional rehabilitation to alleviate her psychological and physical symptoms. These mechanisms have been shown to be some of the most effective components of psychosocial interventions in cancer patients (11).

In order to target the change objectives, we use the learning and communication tools included in the approaches of PROMs and nurse navigation. Figure 1 illustrates the intervention components. With PROMs, we indirectly seek to change patient behaviour, as the PROMs give the nurse navigator information on the symptoms to be targeted in the navigation session and also make the patients aware of the severity of their symptoms. The aim of the nurse navigation sessions is to change social-cognitive factors by e.g. psycho-education (providing knowledge), skills training (improving self-efficacy and problem-solving) and referral to a municipality rehabilitation center for improving self-efficacy. Also, strategies from cognitive behavioural therapy are applied (12). Through counseling and dialogue, nurse navigation aims to build new skills to identify and challenge negative thoughts, reframe negative false assumptions concerning herself and the world, and construct a positive approach. This may aid the process of health behavior change, generating healthy, effective coping skills and assertiveness. A previous study on psychotherapy has estimated the effect of the alliance between the patient and counselor to be up to 54% (12).

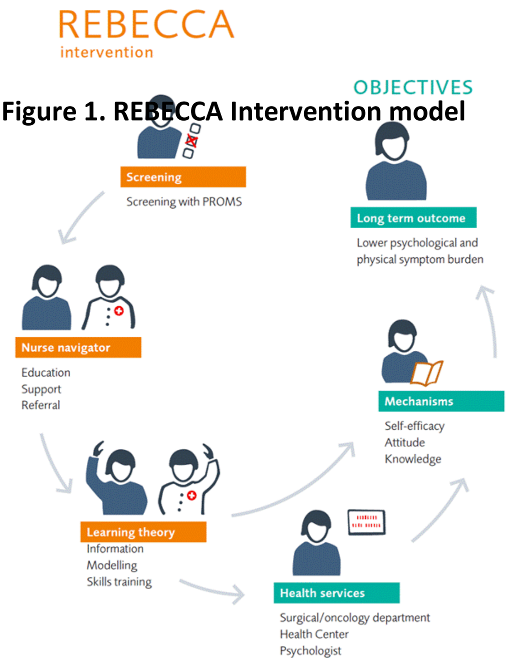

Figure 1. REBECCA Intervention model

Patient reported outcome measures

PROMs screening for psychological and physical symptoms will be performed before surgery (T1) and 1 week (T2), 2 months (T3) and 4 months (T4) after surgery. A positive PROMs screening includes above-normal scores on the distress thermometer or the Functional Assessment of Cancer Therapy –Breast (FACT-B) symptom inventory at any of the four assessments or indicating health behaviour that is not in accordance with recommendations (daily smoking, alcohol consumption of ≥ 7 units per week, body mass index ≥ 25 or physical inactivity < 150 min/week + 20 min intensive recreational training x 2/week) at baseline only (Appendix 7). A positive screening will inform the nurse navigator and the dialogue between the patient and the nurse navigator. However, no fixed actions will be taken based on a positive screening. The PROM symptoms will be reported through the project electronic platform. The results of the PROMs will be available to the nurse where symptoms are categorized as a traffic light with green indicating low symptoms, yellow indicating moderate symptoms with nurse evaluating is necessary and red light indicating immediate nurse evaluation.

Nurse navigation

Nurse navigation includes individual counseling and psycho-education (e.g understanding the cancer diagnosis and the treatment) to motivate and support patients in self-managing their symptoms, and, if relevant, use existing rehabilitation services at the hospital (surgery,

oncology, radiotherapy departments) or at the local rehabilitation centers. In Denmark, cancer rehabilitation is provided by the local municipalities' rehabilitation centers with great variations in terms of rehabilitation services. The nurse navigator will advise the patient on the different opportunities and referral could e.g. be to an oncology nurse for management of pain or nausea, referral to the local health center for management of psychological distress in support groups or for physical exercise to alleviate arm and shoulder problems (Appendix 8). The nurse navigator may furthermore refer the patient to up to six project-funded individual sessions with a project psychologist.

Up to six individual, manual-based, face-to-face or telephone sessions are offered, depending on individual needs (Appendix 9). Each session includes: 1) emphatic listening and dialogue, 2) collaborative empiricism (patient's private world and navigator's professional standards), 3) thorough assessment of needs, 4) psychoeducation, 5) goal setting, 6) intervention –agreements and plan, 7) debriefing and 8) session evaluation using the Session Rating Scale SRS based on Feed-back Informed Treatment. Two nurses (BGM and to-be-named) will be trained to function as nurse navigators. We have, based on experiences from the pilot study decided to apply the instrument Session Rating Scale (SRS) (13) developed in The Client Directed Outcome Informed (CDOI) psychotherapy approach to identify patient tracks with no or negative improvement in order to optimally adjust the care to patient resources, goals, ideas for change and experience of the care collaboration (Appendix 9).

Control group

Patients in the control group receive standard treatment and care, which includes nursing in the hospital departments of surgery, oncology and radiotherapy and referral and access to the local health centers. Nurses in the hospital departments provide care during usual appointments, without systematic symptom screening. Usual care for most patients receiving chemotherapy includes seven nurse appointments (three by telephone) at the surgical department and six to eight appointments at the oncology department. Care may include counselling, psychosocial support, pain management, prevention of nausea and vomiting, temporary breast prosthesis and information.

DATA COLLECTION

Questionanires

Both the intervention and the control group will be assessed before surgery (T_1) and 1 week (T_2), 6 months (T_5) and 12 months (T_6) and 18 months (T_7) after surgery. Patients randomized to the intervention group will also be assessed at 2 months (T_3) and 4 months (T_4). Patients who were not distressed (score < 7 on the distress thermometer) at T_1 will be excluded from the trial but will be followed longitudinally in a sub-study by filling in questionnaires and with the same follow-up assessments as the control group. The questionnaires allow assessment of the primary and secondary outcomes and also covariates and mechanisms. The baseline questionnaire will be paper-based and filled out in the clinic. The follow-up questionnaires will be electronic and a secured link will be sent to the patient's e-mail address.
The electronic platform will manage questionnaire invitations and reminders.

National registries, clinical database and medical records

Clinical, prognostic and treatment factors will be obtained from the Danish Breast Cancer Cooperative Group (14). In order to develop cost-utility analyses, use of health services

including all outpatient visits to any healthcare clinic will be obtained from the local municipality rehabilitation centers, the Danish National Patient Registry (15) and medical records while information on disability and productivity loss (sick leave, disability pension and retirement pension) will be obtained from the Integrated Database for Labor Market Research (16). Further, information on national health service cost will be obtained from DRG costs from Statens Serum Institute. We will obtain information on disease characteristics (stage and tumor characteristics) and treatment (hospitalization, treatment type, municipality-based rehabilitation and dates for these) on their breast cancer as well as other diseases (diagnosis and date).

Outcomes

Data collection instruments used to measure the individual outcomes are illustrated in Figure 2 and items for all questionnaires are shown in Appendix 10. The primary outcome is psychological distress, defined as a multifactorial, unpleasant psychological (cognitive, behavioural, emotional), social or spiritual emotional experience that potentially influences the ability to manage the cancer disease, symptoms and treatment. This will be measured using the distress thermometer. Secondary outcomes include anxiety and depression, breast cancer specific psychological and physical symptoms, quality of life measure to obtain cost-utility, health behaviour (smoking, alcohol consumption, body mass index and physical activity), fear of recurrence, social support, unmet needs, and acceptability. Feasibility outcomes will include indicators of whether the intervention has had an effect on the mechanisms and will include patient activation.

Figure 2. Data collection instruments

Outcome	Scale	Baseline before surgery	3 week screening	10 week screeening	18 week screeening	6 months follow-up	12 months follow-up	18 months follow-up
Demographics	Single items	X						
Social support	MOS	X						
Distress	DT	X	X	X	X	X	X	X
Anxiety	GAD 7	X	X	X	X	X	X	X
Depression	PHQ 9	X	X	X	X	X	X	X
Symptoms	FACT-B	X	X	X	X	X	X	X
Physical function	Physical function scale	X	X	X	X	X		X
Self-efficacy	PAM	X				X	X	X
Fear of recurrence	CARQ					X	X	X
Pain	Single items	X				X	X	X
Neuropathy	Single items	X				X	X	X

**Sleep**	PSQI	X				X	X	X
**Cognitive function**	FACT-cog	X				X	X	X
**Health behavior**	Single items	X						X
**Need for support**	Single items	X				X	X	X
**Quality of life**	**EQ-5D**	X						X
**Acceptibility**	**Single items**							X

Note. MOS Medical Outcomes Study Social Support Survey (17). DT Distress thermometer (18,19). GAD 7 Generalized Anxiety Disorder (20). PHQ 9 (depression) The Patient Health Questionnaire (21). FACT-B Functional Assessment of Cancer Therapy –Breast (22). Physical Function Scale (29). PAM Patient Activation Measure (23). CARQ Fear of Recurrence (24). Pain (single items), neuropathy (single items). PSQI Pittsburgh Sleep Quality Index (25). FACT-cog Functional Assessment of Cancer Therapy-Cognitive (26). Health behavior including smoking, alcohol, physical activity, BMI (single items). Need for support (single items). EQ-5D Euro Quality of Life-5-Dimensional Classification (27).

STATISTICAL ANALYSES

Descriptive analyses will provide information on inclusion, feasibility, including participation in intervention components and data collection, satisfaction with the intervention, and information on nurse management of symptoms from medical records. Analyses will be based on intention-to-treat, with t tests to compare mean differences in change in each group between baseline and 6, 12 and 18 months of follow-up and with mixed models including all follow-ups. Primary analyses will test the effect of the intervention on distress, and secondary analyses will test for psychological symptoms, physical symptoms, and health behaviour (smoking, alcohol, BMI, physical activity). The effect of the intervention in the control and the intervention group will be assumed to be normally distributed and will be tested by the F-test. In all analyses, 99% confidence intervals will be calculated. Analyses will be performed based on the intention to treat principle including all participants and in sensitivity analyses, imputations of missing data will be performed using the last-observation-carried-forward method (28). In analyses aiming to describe the clinical impact of the intervention, we will estimate how many percent of the cases of distress, anxiety, depression and physical symptoms in the intervention versus the control group will be moved to non-cases. Furthermore, in cost-utility analyses, we will apply quality adjusted life years (QALY) to weigh the life expectancy of a patient against the Euro Quality of Life 5-Dimensions Classification (5Q-5D). Using incremental analyses, mean differences in costs (health care +disability + productivity loss + intervention) are compared with mean differences in health consequences for the intervention versus the control group.

Power and participation

We have estimated power on the outcomes: distress (distress thermometer), anxiety and depression (hospital anxiety and depression scale) based on results from the pilot study (N=50) (Figure 3) where we observed an up to 30% difference in change from case to non-case in the intervention versus the control group on distress and anxiety.

Figure 3. Percentage of women changing from cases to non-cases in intervention and control group

	Control group (%)	Intervention group (%)

**Distress (≥ 7)**	61	90
**Anxiety (≥ 8)**	53	80
**Depression (≥ 8)**	44	49

Conservatively, we expect slightly lowered differences in change from case to non-case in the large full-scale study (20 %), compared to those observed in the pilot study (30%).

In a 2-year period, we expect 948 women to fulfill the inclusion criteria. With an expected participation rate of 80% (85% was reach in the pilot study) 758 will participate. Of these 43% will have high distress (N=324) and will enter the RCT. 57% will have low distress (n=432) and will be invited to participate in observation group who will fill-out questionnaires.

Due to a large number of planned analyses in the RCT study, we assume a highly conservative alfa to be 1%. Within a 2-year inclusion period, we will include 324 patients and an estimated 20% will drop-out or have missing data, resulting in final sample of 260.

With 130 women in each group, an expected 61% women move from cases to non-cases on distress in the control group and 81% percent women move from cases to non-cases on distress in the intervention group, we will obtain 84% power. Similar calculations for anxiety and depression reveals power of 78% and 76%.

ORGANIZATION

Christoffer Johansen, Professor, Ph.D., DMSc will be the principle investigator of the project. The daily project coordinator will be Birgitte Goldschmidt Mertz, Nurse specialist, R.N. and Pernille Bidstrup, Senior Researcher, Psychologist, Ph.D. will be research coordinator. The project will be followed by an advisory board including clinicians and researchers with great expertise in breast cancer patients.

The main project group includes:

- The Danish Cancer Society Research Center
  - Pernille Bidstrup, Christoffer Johansen
- Section of Breast Surgery, Rigshospitalet
  - Birgitte Goldschmidt Mertz, Niels Kroman,

Further, the study advisory board includes:

- Oncology Clinic, Rigshospitalet
  - Michael Andersson, Ulla Breitenstein Mathiesen
- Oncology Clinic, Herlev Hospital
  - Gosia Tuxen
- Copenhagen Centre for Cancer and Health
  - Jette Vibe-Petersen
- The Danish Cancer Society Research Center
  - Susanne Dalton
- Section for surgical pathophysiology, Rigshospitalet
  - Kenneth Geving Andersen

The project will start in April 2017, inclusion will end in April 2019, follow-up will end in October 2021 and analyses will end in October 2023.

ETHICS

The study will follow ethical procedures for health research in Denmark, which is in accordance with recommendations from the National Board of Data Protection, ethical standards as outlined by the The Capital Region of Denmark's Ethical Committee ensuring voluntary participation and the right to best treatment available, irrespective of study participation. We will apply the Capital Region of Denmark's Board of Data Protection for permission. The study will also follow recommendations by the Helsinki-declaration II, the SPIRIT and CONSORT for randomized controlled trials of non-pharmacological treatment. The RCT will be made public, including the protocols at ClinicalTrials.gov before inclusion of the first participant.

Participation will be voluntary. Patients will receive written and verbal information about the study and will have 24 hours consideration period before signing informed consent form. Participants have the right to withdraw from the study at any time without giving any reason and withdrawal has no consequences for the participants continued treatment. We will collect information from national registries and from medical records and for this purpose we will obtain informed consent according to the Danish Health Legislation paragraph 43.1.

Participants in the REBECCA study will not be restricted from any activities or treatments outside the study. Inclusion in the study in either the intervention or the control arm do does not involve any known side-effects, risks or harm. Further to ensure that participants will experience as few inconveniences as possible related to filling out the questionnaires we will use validate scales, which have already been tested on larger populations. We cannot role out that the intervention may lead to increased risk for uncertainty and anxiety. All participants in the study are instructed to report if they experience any side effects, risks or harms associated with study participation. The participants in this study are covered by "The Danish Patient Insurance" although some of the trial investigators are not employed at a hospital.

There are no known circumstances that may lead to disruption of this study or that participants will be excluded from study participation. If this, contrary to expectations should be the case, participants will be informed about the reason for this. Participants who no longer fulfill the inclusion criteria, will not be excluded from the study. Patients will only be excluded from the study if they at any time do not wish to participate.

Data collected for this study will be securely stored according to the requirements from The Danish Data Protection Board at The Danish Cancer Society Research Center and at Section of Breast Surgery, Rigshospitalet. Permission for data storage is obtained from Region Hovedstaden.

DISEMMINATION

The results of the study will be disseminated through at least four scientific peer-reviewed publications. Further, a short Danish report will be developed summarizing the results and the clinical implications. We wish to ensure that knowledge from the project reach as far as possible to health professionals at hospitals, municipalities and private organizations

offering volunteer support. Results from the project will be presented at the yearly Danish breast cancer workshop targeting all nurses and medical doctors in Demark and at two international conferences. Finally, a seminar will be arranged to present and discuss the results of the study. Here, we will invite health professionals in Denmark working with cancer rehabilitation, politicians and administrators from the health area as well as patients and relatives.

Working titles of peer-reviewed publications
Paper 1 RCT on the effect of individually tailored nurse navigation for distressed breast cancer patients on distress, anxiety and depression
Paper 2 RCT on the effect of individually tailored nurse navigation for distressed breast cancer patients on secondary outcomes of physical symptoms
Paper 3 The mechanisms at play in nurse navigation
Paper 4 The price for nurse navigation: Evaluation of an RCT comparing the cost of nurse-navigation and standard care

PERSPECTIVES
The screening procedure proposed for eligibility is very simple and includes the single-item distress thermometer. It is expected that this will provide optimal acceptability from health professionals and could be implemented in clinical cancer settings with few barriers. The screening procedure applied at four time points is also developed to be as simple as possible. FACT-B and the distress thermometer. Still, implementation of the intervention into clinical practice will demand nurse resources to manage the screening procedure and the symptoms identified. This underlines the importance of our planned evaluation of implementation cost and cost-effectiveness as part of the study design as this will inform hospitals in Denmark of the pros and cons of the intervention.

If the REBECCA intervention shows positive effects on psychological and physical symptoms both in terms of statistical and clinical significance and if the cost-effectiveness is promising, we will contact all breast cancer hospital departments and hospital owners in the Danish regions to offer support on any implementation processes e.g. by providing the nurse navigation manual and training in its use.

FUNDING
The Tryg Foundation has supported this project. The investigators are independent of Trygfonden and Trygfonden will have no influence on the research process or interpretation of results. The funding will be distributed equally during the five-year study period and the funders are responsible for the current audit of each their account. Salary to the project staff will be covered from these accounts. No financial reimbursement will be given to BC patients for participation in this study.

REFERENCES

1. Reilly, C.M., et al., A literature synthesis of symptom prevalence and severity in persons receiving active cancer treatment. Support Care Cancer, 2013. 21(6): p. 1525-50.
2. Bidstrup, P.E., et al., Trajectories of distress, anxiety, and depression among women with breast cancer: Looking beyond the mean. Acta Oncol, 2015. 54(5): p. 789-96.
3. von Heymann-Horan, A.B., et al., Unmet needs of women with breast cancer during and after primary treatment: a prospective study in Denmark. Acta Oncol, 2013. 52(2): p. 382-90.
4. Wagner, E.H., et al., The quality of cancer patient experience: perspectives of patients, family members, providers and experts. Qual Saf Health Care, 2010. 19(6): p. 484-9.
5. Berry, D.L., et al., Enhancing patient-provider communication with the electronic self-report assessment for cancer: a randomized trial. J Clin Oncol, 2011. 29(8): p. 1029-35.
6. Kotronoulas G, Kearney N, Maguire R, et al.: What is the value of the routine use of patient-reported outcome measures toward improvement of patient outcomes, processes of care, and health service outcomes in cancer care? A systematic review of controlled trials. J Clin Oncol 2014; 32:1480-1501.
7. Wagner EH, Ludman EJ, Aiello Bowles EJ, et al.: Nurse navigators in early cancer care: a randomized, controlled trial. J Clin Oncol 2014; 32:12-18.
8. Skrutkowski, M., et al., Impact of a pivot nurse in oncology on patients with lung or breast cancer: symptom distress, fatigue, quality of life, and use of healthcare resources. Oncol Nurs Forum, 2008. 35(6): p. 948-54.
9. Fiscella, K., et al., Patient navigation for breast and colorectal cancer treatment: a randomized trial. Cancer Epidemiol Biomarkers Prev, 2012. 21(10): p. 1673-81.
10. Envold BP, Mertz BG, Kroman N, et al.: Tailored nurse navigation for women treated for breast cancer: Design and rationale for a pilot randomized controlled trial. Acta Oncol 2016;1-5.
11. Stanton, A.L., et al., Mechanisms in psychosocial interventions for adults living with cancer: opportunity for integration of theory, research, and practice. J Consult Clin Psychol, 2013. 81(2): p. 318-35.
12. Wampold, B.E., The great psychotherapy debate: Models, methods, and findings. 2001, Hillsdale N.J.: Lawrence Erlbaum.
13. Duncan, B.L., et al., The Session Rating Scale: Preliminary Psychometric Properties of a “Working” Alliance Measure. Journal of Brief Therapy, 2003. 3(1): p. 8.
14. Moller, S., et al., The clinical database and the treatment guidelines of the Danish Breast Cancer Cooperative Group (DBCG); its 30-years experience and future promise. Acta Oncol, 2008. 47(4): p. 506-24.
15. Lynge, E., et al. The Danish National Patient Register. Scand J Public Health, 2011. 39(7 Suppl): p. 30-3.
16. Baadsgaard, M. et al. Danish registers on personal income and transfer payments. Scand J Public Health, 2011. 39(7 Suppl): p. 103-5.
17. Moser, A., et al., The eight-item modified Medical Outcomes Study Social Support Survey: psychometric evaluation showed excellent performance. J Clin Epidemiol, 2012. 65(10): p. 1107-16.
18. Roth AJ et al.: Rapid screening for psychologic distress in men with prostate carcinoma: a pilot study. Cancer 1998; 82:1904-1908.

19. Bidstrup PE et al.: Accuracy of the Danish version of the 'distress thermometer'. Psychooncology 2011; 21:436-443
20. Spitzer, R.L. et al., A brief measure for assessing generalized anxiety disorder: the GAD-7. Arch Intern Med, 2006. 166(10): p. 1092-7.
21. Kroenke, K. et al. The PHQ-9. Validity of a Brief Depression Severity Measure. J Gen Intern Med., 2001. 16(9): p. 7.
22. Brady MJ et al. Reliability and validity of the Functional Assessment of Cancer Therapy-Breast quality-of-life instrument. Journal of clinical oncology : official journal of the American Society of Clinical Oncology. 1997;15(3):974-86.
23. Hibbard JH et al. Development of the Patient Activation Measure (PAM): conceptualizing and measuring activation in patients and consumers. Health services research. 2004;39(4 Pt 1):1005-26.
24. Thewes B et al. The Concerns About Recurrence Questionnaire: validation of a brief measure of fear of cancer recurrence amongst Danish and Australian breast cancer survivors. Journal of cancer survivorship : research and practice. 2015;9(1):68-79.
25. Buysse, et al. The Pittsburgh Sleep Quality Index (PSQI): A new instrument for psychiatric research and practice. Psychiatry Research, 1989; 28(2), 193-213

26. Von Ah at al. Perceived cognitive function in breast cancer survivors: evaluating relationships with objective cognitive performance and other symptoms using the functional assessment of cancer therapy-cognitive function instrument. J Pain Symptom Manage. 2015 Apr;49(4):697-706.
27. EuroQol--a new facility for the measurement of health-related quality of life. Health Policy, 1990. 16(3): p. 199-208.
28. Shao, J. and B. Zhong, Last observation carry-forward and last observation analysis. Stat Med, 2003. 22(15): p. 2429-41.
29. Andersen KG et al. The Effect of Pain on Physical Functioning after Breast Cancer Treatment: Development and Validation of an Assessment Tool. Clin J Pain, 2014. [Epub ahead of print].
