# Supplementary material for: Effect of a Nurse Navigation Intervention on Mental Symptoms in Patients With Psychological Vulnerability and Breast Cancer: The REBECCA Randomized Clinical Trial
Source: JAMA Netw Open. 2023 Jun 23;6(6):e2319591. doi: 10.1001/jamanetworkopen.2023.19591 (PMC10290249; doi:10.1001/jamanetworkopen.2023.19591)
Supplement: Supplement 2. — eTable 1. Need for Support and Health Behaviour Outcomes at 18 Months Follow-up in Patients With Breast Cancer in the REBECCA Study eTable 2. Primary and Secondary Outcomes Using Multiple Imputations at Random and Not at Random at 6-, 12-, and 18-Months Follow-up in 309 Patients With Breast Cancer in the REBECCA Study eFigure 1. Schedule of Enrollment, Interventions and Assessment in the REBECCA Randomized Controlled Trial eFigure 2. Standardized Interaction Effects for Distress at 6, 12, and 18 Months Follow-up in 309 Patients With Breast Cancer in the REBECCA Study eFigure 3. Standardized Interaction Effects for Anxiety at 6, 12, and 18 Months Follow-up in 309 Patients With Breast Cancer in the REBECCA Study eFigure 4. Standardized Interaction Effects for Depression at 6, 12, and 18 Months Follow-up in 309 Patients With Breast Cancer in the REBECCA Study eFigure 5. Standardized Interaction Effects for Breast Cancer-Specific HQoL at 6, 12, and 18 Months Follow-up in 309 Patients With Breast Cancer in the REBECCA Study eFigure 6. Standardized Interaction Effects for Sleep Function at 6, 12, and 18 Months Follow-up in 309 Patients With Breast Cancer in the REBECCA Study eFigure 7. Standardized Interaction Effects for Fear of Recurrence at 6, 12, and 18 Months Follow-up in 309 Patients With Breast Cancer in the REBECCA Study eFigure 8. Standardized Interaction Effects for Patient Activation at 6, 12, and 18 Months Follow-up in 309 Patients With Breast Cancer in the REBECCA Study eFigure 9. Standardized Interaction Effects for Cognitive Function at 6, 12, and 18 Months Follow-up in 309 Patients With Breast Cancer in the REBECCA Study [file jamanetwopen-e2319591-s002.pdf]

Supplemental Online Content

Bidstrup PE, Johansen C, Kroman N, et al. Effect of a nurse navigation intervention on mental symptoms in patients with psychological vulnerability and breast cancer: the REBECCA randomized clinical trial. *JAMA Netw Open*. 2023;6(6):e2319591. doi:10.1001/jamanetworkopen.2023.19591

eTable 1. Need for Support and Health Behaviour Outcomes at 18 Months Follow-up in Patients With Breast Cancer in the REBECCA Study
eTable 2. Primary and Secondary Outcomes Using Multiple Imputations at Random and Not at Random at 6-, 12-, and 18-Months Follow-up in 309 Patients With Breast Cancer in the REBECCA Study
eFigure 1. Schedule of Enrollment, Interventions and Assessment in the REBECCA Randomized Controlled Trial
eFigure 2. Standardized Interaction Effects for Distress at 6, 12, and 18 Months Follow-up in 309 Patients With Breast Cancer in the REBECCA Study
eFigure 3. Standardized Interaction Effects for Anxiety at 6, 12, and 18 Months Follow-up in 309 Patients With Breast Cancer in the REBECCA Study
eFigure 4. Standardized Interaction Effects for Depression at 6, 12, and 18 Months Follow-up in 309 Patients With Breast Cancer in the REBECCA Study
eFigure 5. Standardized Interaction Effects for Breast Cancer-Specific HQoL at 6, 12, and 18 Months Follow-up in 309 Patients With Breast Cancer in the REBECCA Study
eFigure 6. Standardized Interaction Effects for Sleep Function at 6, 12, and 18 Months Follow-up in 309 Patients With Breast Cancer in the REBECCA Study
eFigure 7. Standardized Interaction Effects for Fear of Recurrence at 6, 12, and 18 Months Follow-up in 309 Patients With Breast Cancer in the REBECCA Study
eFigure 8. Standardized Interaction Effects for Patient Activation at 6, 12, and 18 Months Follow-up in 309 Patients With Breast Cancer in the REBECCA Study
eFigure 9. Standardized Interaction Effects for Cognitive Function at 6, 12, and 18 Months Follow-up in 309 Patients With Breast Cancer in the REBECCA Study

This supplemental material has been provided by the authors to give readers additional information about their work.

© 2023 Bidstrup PE et al. *JAMA Network Open.*

eTable 1. Need for Support and Health Behaviour Outcomes at 18 Months Follow-up in Patients With Breast Cancer in the REBECCA Study

	N	OR	CI	p-value
**Unmet need for**				
Physical problems	45	0.79	0.13;4.90	0.80
Family problems	47	0.44	0.08;2.48	0.35
Psychological support	117	0.89	0.33;2.38	0.81
Work problems	70	2.30	0.63;8.48	0.21
Economic problems	47	0.13	0.01;1.59	0.11
**Health behavior**				
No current smoking	241	1.11	0.31;3.87	0.88
Drink less than once per week	239	1.87	0.77;4.50	0.16
Drink below 7 drinks	98	0.58	0.23;.147	0.25
More physically active	242	0.97	0.58;1.61	0.90

© 2023 Bidstrup PE et al. *JAMA Network Open.*

eTable 2. Primary and Secondary Outcomes Using Multiple Imputations at Random and Not at Random at 6-, 12-, and 18-Months Follow-up in 309 Patients With Breast Cancer in the REBECCA Study

Intervention effect	Linear mixed model missing at random[a]			Linear mixed model missing not at random[b]		
	Estimate	95% CI	P	Estimate [c]	95% CI	P
Distress ♦						
6 months	-0.51	-1.08;0.06	0.08	-0.56	-1.17;0.05	0.07
12 months	-0.56	-1.14;0.02	0.06	-0.63	-1.25;0.00	0.05
18 months	-0.11	-0.71;0.49	0.72	-0.13	-0.79;0.52	0.68
Anxiety ♦						
6 months	**-1.01**	**-1.95-0.06**	**0.04**	**-1.09**	**-2.08;-0.09**	**0.03**
12 months	**-1.03**	**-1.97;-0.10**	**0.03**	**-1.15**	**-2.12;-0.17**	**0.02**
18 months	-0.90	-1.90;0.10	0.08	-0.95	-2.02;0.12	0.08
Depression ♦						
6 months	**-1.34**	**-2.30; -0.38**	**0.01**	**-1.41**	**-2.42; -0.40**	**0.01**
12 months	-0.62	-1.56; 0.31	0.19	-0.73	-1.71; 0.24	0.14
18 months	-0.30	-1.29; 0.69	0.56	-0.33	-1.38; 0.73	0.54
HQoL ◊						
6 months	2.36	-0.46; 5.18	0.10	2.57	-0.25; 5.39	0.07
12 months	**4.14**	**1.34; 6.93**	**0.00**	**4.59**	**1.79; 7.40**	**0.00**

© 2023 Bidstrup PE et al. *JAMA Network Open.*

18 months	2.18	-0.66; 5.03	0.13	2.05	-0.79; 4.88	0.16
Sleep function ♦						
6 months	-0.29	-1.07; 0.49	0.47	-0.38	-1.22; 0.47	0.38
12 months	0.09	-0.71; 0.89	0.83	-0.00	-0.87; 0.86	0.99
18 months	0.02	-0.80; 0.83	0.97	0.02	-0.86; 0.90	0.96
Fear of recurrence ♦[c]						
6 months	-1.95	-4.40; 0.50	0.12	-2.18	-4.81; 0.44	0.10
12 months	-2.23	-4.65; 020	0.07	-2.48	-5.07; 0.11	0.06
18 months	-1.67	-4.18; 0.85	0.19	-1.77	-4.49; 0.94	0.20
Patient activation ◊						
6 months	1.02	-2.39;4.42	0.56	2.21	-1.08;5.50	0.19
12 months	3.02	-0.62;6.66	0.10	**4.19**	**0.75;7.64**	**0.02**
18 months	3.64	-0.13;7.41	0.06	**4.33**	**0.80;7.86**	**0.02**
Cognitive function ◊						
6 months	2.55	-0.16; 5.25	0.06	**2.84**	**0.14; 5.53**	**0.02**
12 months	1.95	-0.88; 4.78	0.18	2.38	-0.39; 5.16	0.09
18 months	1.57	-1.22; 4.35	0.27	1.71	-1.03; 4.46	0.22

Models were adjusted for randomization strata (age (< 60; ≥ 60 years) and treatment modality (no or adjuvant chemotherapy; neo-adjuvant).

[a] Assuming that data were missing at random.

[b] Assuming that those with missing data would have 20% worse symptoms than expected.

© 2023 Bidstrup PE et al. *JAMA Network Open.*

[c] No baseline level to take into account.

♦ Higher score indicating higher symptoms

◊ Higher score indicating better quality of life/activation/function

© 2023 Bidstrup PE et al. *JAMA Network Open.*

eFigure 1. Schedule of Enrollment, Interventions and Assessment in the REBECCA Randomized Controlled Trial

	STUDY PERIOD											
	**Enrolment**	**Allocation**	**Intervention**							**Follow-up (FU)**		
**TIMEPOINT**	***$-t_1$***	**0**	***T_1***	***T_2***	***T_3***	***T_4***	***T_5***	***T_6***	***$T_{7\text{-}9}$***	***FU_6 months***	***FU_{12} months***	***FU_{18} months***
**ENROLMENT:**	-1	0										
Eligibility screen	X											
Informed consent	X											
Distress screen	X											
Allocation		X										
**INTERVENTIONS:**												
REBECCA			◆——	——	——	——	——	——	——◆			
Control												
**ASSESSMENTS:**												
Sociodemographic information	X											
Social support	X											

© 2023 Bidstrup PE et al. *JAMA Network Open.*

Distress	X									X	X	X
Anxiety	X									X	X	X
Depression	X									X	X	X
HQoL	X									X	X	X
Fear of recurrence										X	X	X
Sleep	X									X	X	X
Cognitive function	X									X	X	X
Patient activation	X									X	X	X
Pain	X									X	X	X
Health behavior											X	
Need for support	X										X	
Treatment factors	X									X	X	X
Intervention exposure	X									X	X	X
Patient acceptability											X	

Sociodemographic information (age (<60; >=60 years), education (<12; 12-15; >15 years), employment (not employed; employed), cohabitating partner (no; yes)), social support, Medical Outcomes Study Survey (MOS). Distress, Distress Thermometer; Anxiety, Generalized Anxiety

© 2023 Bidstrup PE et al. *JAMA Network Open.*

Disorder (GAD-7), depression Patient Health Questionnaire (PHQ-9); breast cancer-specific HQoL, Trial Outcome Index-Physical/Functional/Breast (TOI-PFB) score from the Functional Assessment of Cancer Therapy-Breast (FACT-B) scale, fear of recurrence, Concerns About Recurrence Questionnaire (CARQ-4); sleep, Pittsburgh Sleep Quality Index (PSQI), cognitive function, the Functional Assessment of Cancer Therapy-Cognitive (FACT-cog), patient activation, Patient Activation Measure (PAM), pain, Neuropathic Pain scale for Postsurgical patients (NeuPPS), health behavior (smoking, alcohol use, physical activity and BMI), and need for support (single items). Treatment factors including Breast surgery (lumpectomy; mastectomy; mastectomy with primary reconstruction), axillary surgery (axillary dissection; sentinel node biopsy), adjuvant radiotherapy (no; yes), adjuvant endocrine therapy (no; yes), chemotherapy (adjuvant; neoadjuvant; none), trastuzumab (no; yes). In the intervention group we obtained information on intervention exposure (single items) as well as patient reported acceptability (single items) were obtained.

© 2023 Bidstrup PE et al. *JAMA Network Open.*

eFigure 2. Standardized Interaction Effects for Distress at 6, 12, and 18 Months Follow-up in 309 Patients With Breast Cancer in the REBECCA Study

Distress

	Standardized effects (95% CI)
**Age**	**p = 0.045**
**<60**	
6 months	−0.01 (−0.22; 0.19)
12 months	−0.23 (−0.44;−0.03)
18 months	0.00 (−0.21; 0.21)
**>=60**	
6 months	−0.38 (−0.67;−0.10)
12 months	−0.01 (−0.29; 0.28)
18 months	−0.03 (−0.32; 0.26)
**Education**	**p = 0.142**
**<12**	
6 months	−0.41 (−0.78;−0.03)
12 months	−0.40 (−0.77;−0.02)
18 months	−0.38 (−0.78; 0.02)
**12−15**	
6 months	−0.11 (−0.32; 0.09)
12 months	−0.16 (−0.37; 0.05)
18 months	−0.01 (−0.22; 0.21)
**>15**	
6 months	0.08 (−0.34; 0.49)
12 months	0.16 (−0.25; 0.57)
18 months	0.29 (−0.13; 0.72)
**Social support**	**p = 0.359**
**High**	
6 months	−0.14 (−0.32; 0.04)
12 months	−0.18 (−0.37; 0.00)
18 months	−0.01 (−0.21; 0.18)
**Low**	
6 months	−0.22 (−0.64; 0.19)
12 months	−0.10 (−0.51; 0.31)
18 months	−0.15 (−0.57; 0.27)
**Patient activation**	**p = 0.024**
**Low**	
6 months	−0.42 (−0.68;−0.15)
12 months	−0.33 (−0.60;−0.07)
18 months	−0.26 (−0.53; 0.01)
**High**	
6 months	0.02 (−0.35; 0.38)
12 months	0.19 (−0.17; 0.56)
18 months	0.08 (−0.30; 0.46)
**Chemotherapy**	**p = 0.139**
**Adjuvant**	
6 months	−0.25 (−0.49; 0.00)
12 months	−0.37 (−0.61;−0.12)
18 months	−0.16 (−0.42; 0.10)
**Neoadjuvant**	
6 months	0.10 (−0.26; 0.47)
12 months	−0.02 (−0.40; 0.35)
18 months	0.15 (−0.23; 0.53)
**None**	
6 months	−0.20 (−0.48; 0.08)
12 months	−0.01 (−0.29; 0.27)
18 months	0.03 (−0.26; 0.32)

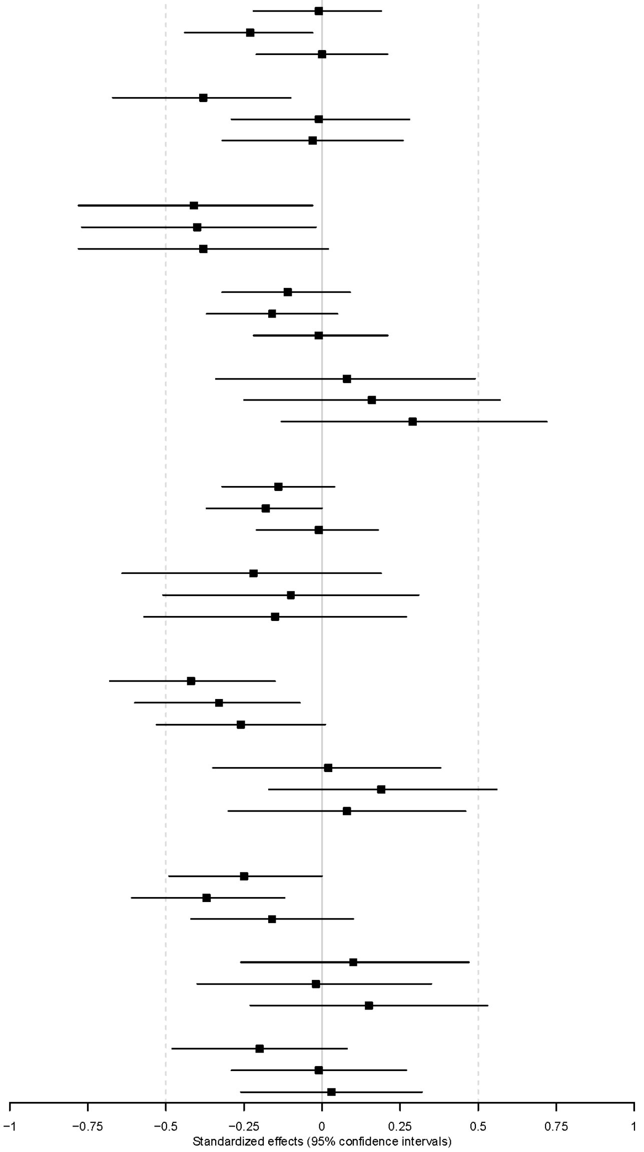

Note. Higher distress score indicates worse distress. Standardized outcome scores were applied using fitted models by subtracting the sample mean score from each score and

© 2023 Bidstrup PE et al. *JAMA Network Open.*

dividing by the standard deviation. Models were adjusted for randomization strata (age (< 60; ≥ 60 years) and treatment modality (no or adjuvant chemotherapy; neo-adjuvant).

© 2023 Bidstrup PE et al. *JAMA Network Open.*

eFigure 3. Standardized Interaction Effects for Anxiety at 6, 12, and 18 Months Follow-up in 309 Patients With Breast Cancer in the REBECCA Study

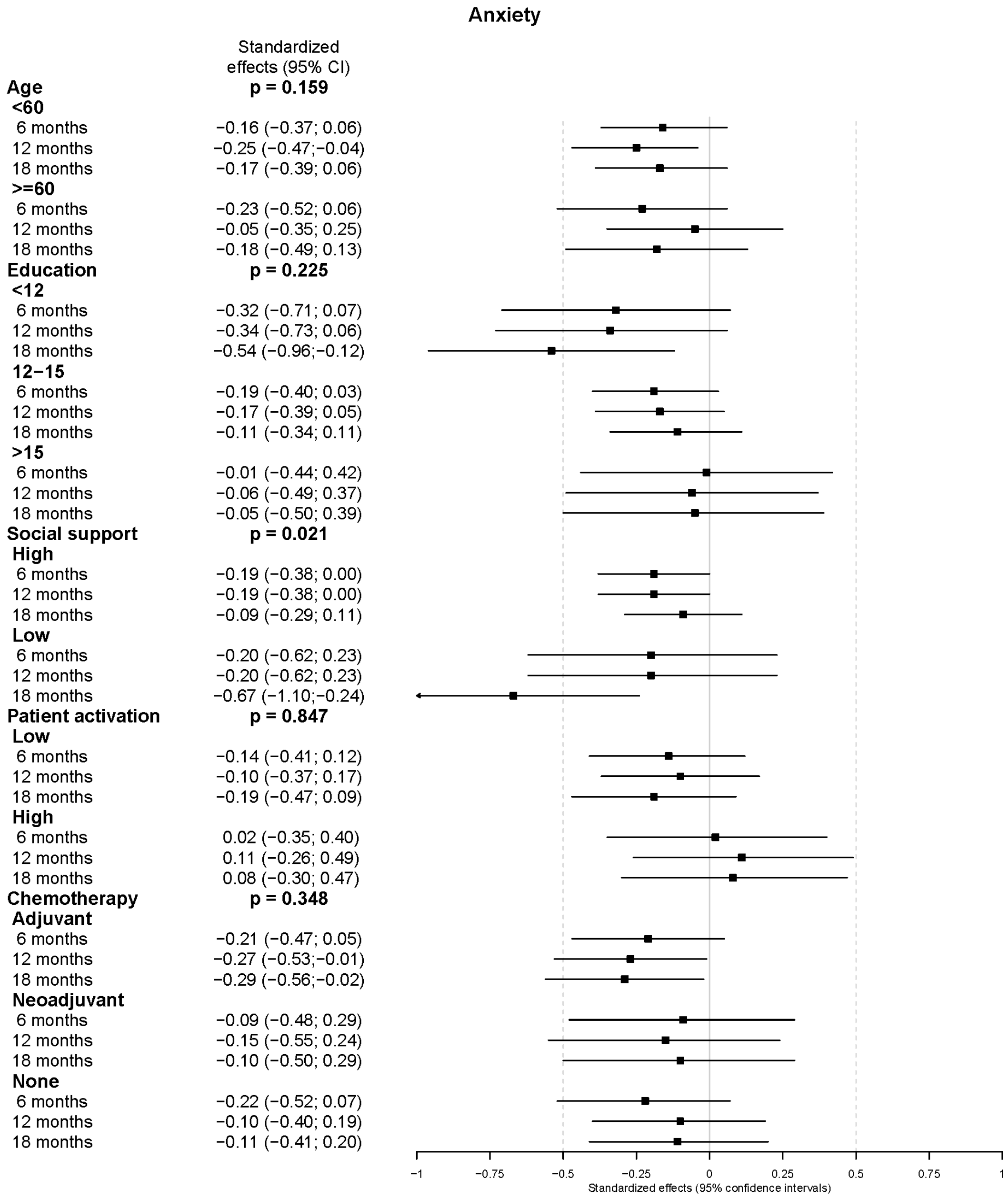

Note. Higher anxiety score indicates worse anxiety. Standardized outcome scores were applied using fitted models by subtracting the sample mean score from each score and

© 2023 Bidstrup PE et al. *JAMA Network Open.*

dividing by the standard deviation. Models were adjusted for randomization strata (age (< 60; ≥ 60 years) and treatment modality (no or adjuvant chemotherapy; neo-adjuvant).

© 2023 Bidstrup PE et al. *JAMA Network Open.*

eFigure 4. Standardized Interaction Effects for Depression at 6, 12, and 18 Months Follow-up in 309 Patients With Breast Cancer in the REBECCA Study

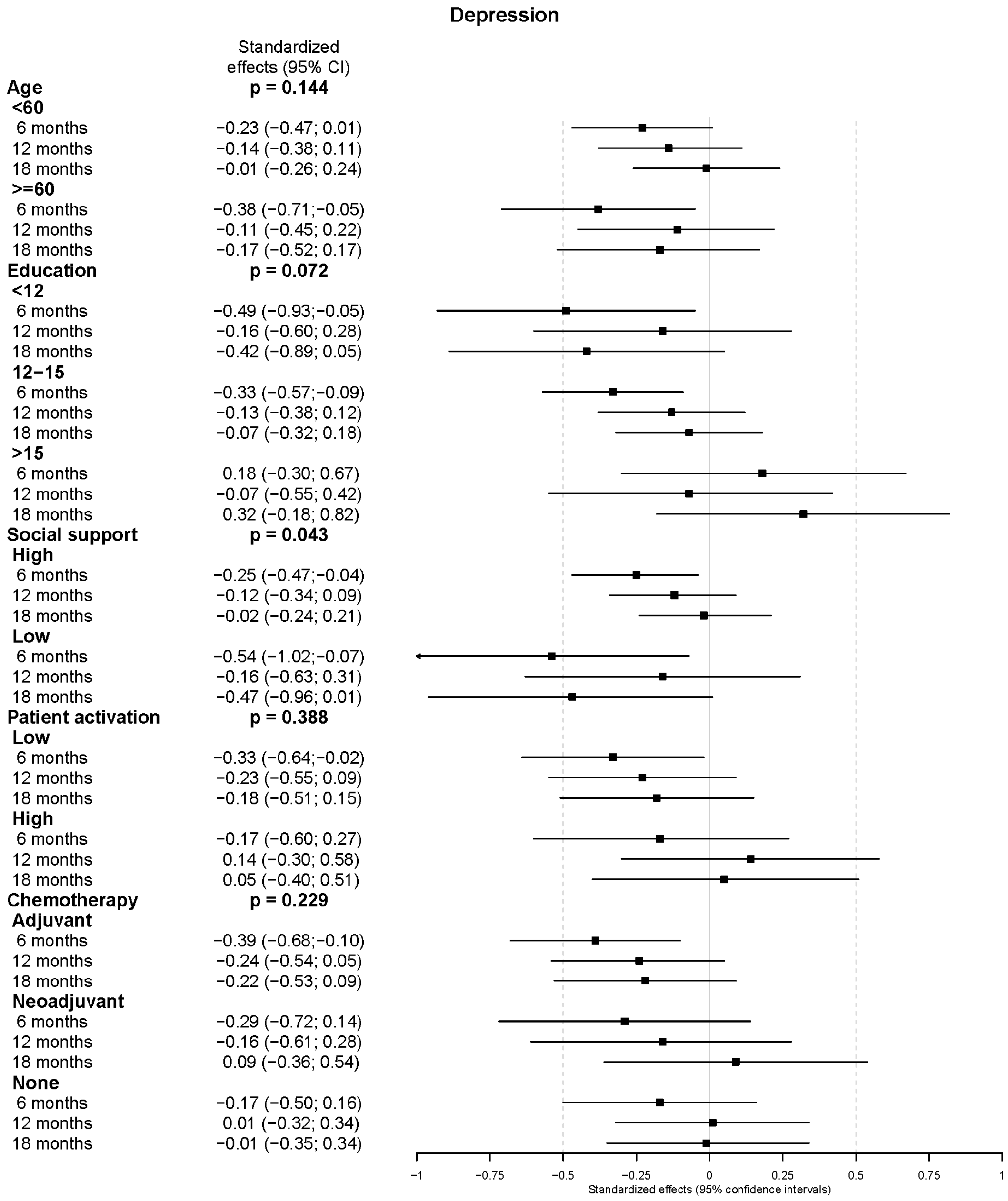

Note. Higher depression score indicates worse depression. Standardized outcome scores were applied using fitted models by subtracting the sample mean score from each score and

© 2023 Bidstrup PE et al. *JAMA Network Open.*

dividing by the standard deviation. Models were adjusted for randomization strata (age (< 60; ≥ 60 years) and treatment modality (no or adjuvant chemotherapy; neo-adjuvant).

© 2023 Bidstrup PE et al. *JAMA Network Open.*

eFigure 5. Standardized Interaction Effects for Breast Cancer-Specific HQoL at 6, 12, and 18 Months Follow-up in 309 Patients With Breast Cancer in the REBECCA Study

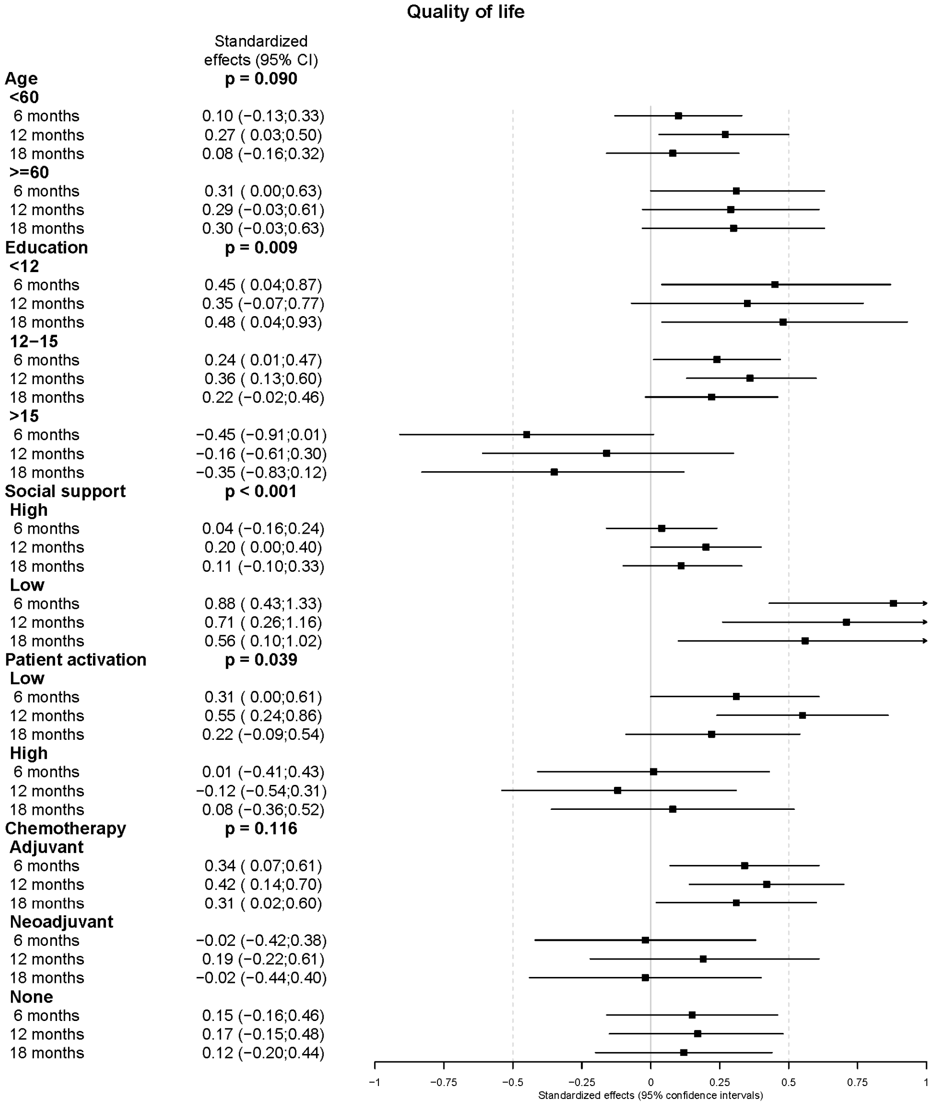

Note. Higher quality of life score indicates better quality of life. Standardized outcome scores were applied using fitted models by subtracting the sample mean score from each score and dividing by the standard deviation. Models were adjusted for randomization strata (age (< 60; ≥ 60 years) and treatment modality (no or adjuvant chemotherapy; neo-adjuvant).

© 2023 Bidstrup PE et al. *JAMA Network Open.*

eFigure 6. Standardized Interaction Effects for Sleep Function at 6, 12, and 18 Months Follow-up in 309 Patients With Breast Cancer in the REBECCA Study

Sleep

	Standardized effects (95% CI)
**Age**	**p = 0.110**
**<60**	
6 months	0.12 (−0.12; 0.36)
12 months	0.08 (−0.15; 0.32)
18 months	0.19 (−0.06; 0.43)
**>=60**	
6 months	−0.41 (−0.74;−0.08)
12 months	−0.08 (−0.41; 0.25)
18 months	−0.34 (−0.68; 0.00)
**Education**	**p = 0.914**
**<12**	
6 months	0.03 (−0.41; 0.48)
12 months	0.03 (−0.41; 0.47)
18 months	0.27 (−0.19; 0.74)
**12−15**	
6 months	−0.14 (−0.38; 0.10)
12 months	0.01 (−0.23; 0.25)
18 months	−0.10 (−0.35; 0.14)
**>15**	
6 months	0.13 (−0.35; 0.60)
12 months	0.13 (−0.34; 0.60)
18 months	0.13 (−0.36; 0.61)
**Social support**	**p = 0.618**
**High**	
6 months	−0.02 (−0.23; 0.19)
12 months	0.11 (−0.11; 0.32)
18 months	0.06 (−0.16; 0.27)
**Low**	
6 months	−0.25 (−0.72; 0.22)
12 months	−0.33 (−0.81; 0.14)
18 months	−0.37 (−0.86; 0.13)
**Patient activation**	**p = 0.544**
**Low**	
6 months	−0.10 (−0.41; 0.21)
12 months	−0.03 (−0.35; 0.28)
18 months	−0.05 (−0.38; 0.27)
**High**	
6 months	−0.17 (−0.62; 0.27)
12 months	0.33 (−0.10; 0.76)
18 months	0.12 (−0.33; 0.58)
**Chemotherapy**	**p = 0.675**
**Adjuvant**	
6 months	−0.19 (−0.48; 0.10)
12 months	−0.04 (−0.33; 0.25)
18 months	−0.08 (−0.38; 0.22)
**Neoadjuvant**	
6 months	0.07 (−0.36; 0.50)
12 months	0.19 (−0.25; 0.62)
18 months	0.42 (−0.02; 0.86)
**None**	
6 months	0.03 (−0.30; 0.36)
12 months	0.03 (−0.30; 0.36)
18 months	−0.15 (−0.49; 0.20)

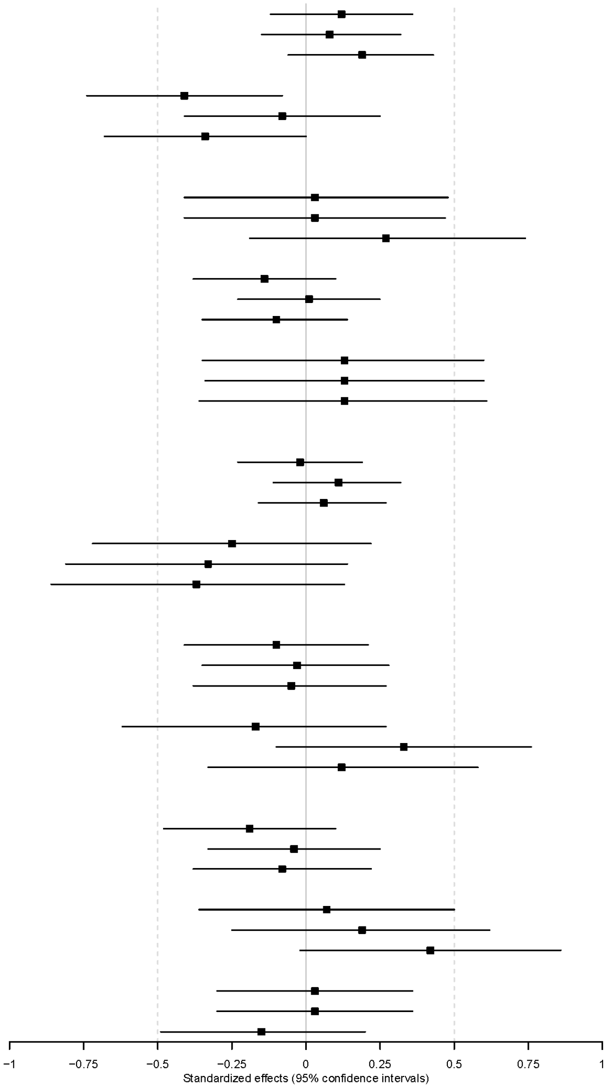

Note. Higher sleep score indicates worse sleep. Standardized outcome scores were applied using fitted models by subtracting the sample mean score from each score and dividing by the standard deviation. Models were adjusted for randomization strata (age (< 60; ≥ 60 years) and treatment modality (no or adjuvant chemotherapy; neo-adjuvant).

© 2023 Bidstrup PE et al. *JAMA Network Open.*

eFigure 7. Standardized Interaction Effects for Fear of Recurrence at 6, 12, and 18 Months Follow-up in 309 Patients With Breast Cancer in the REBECCA Study

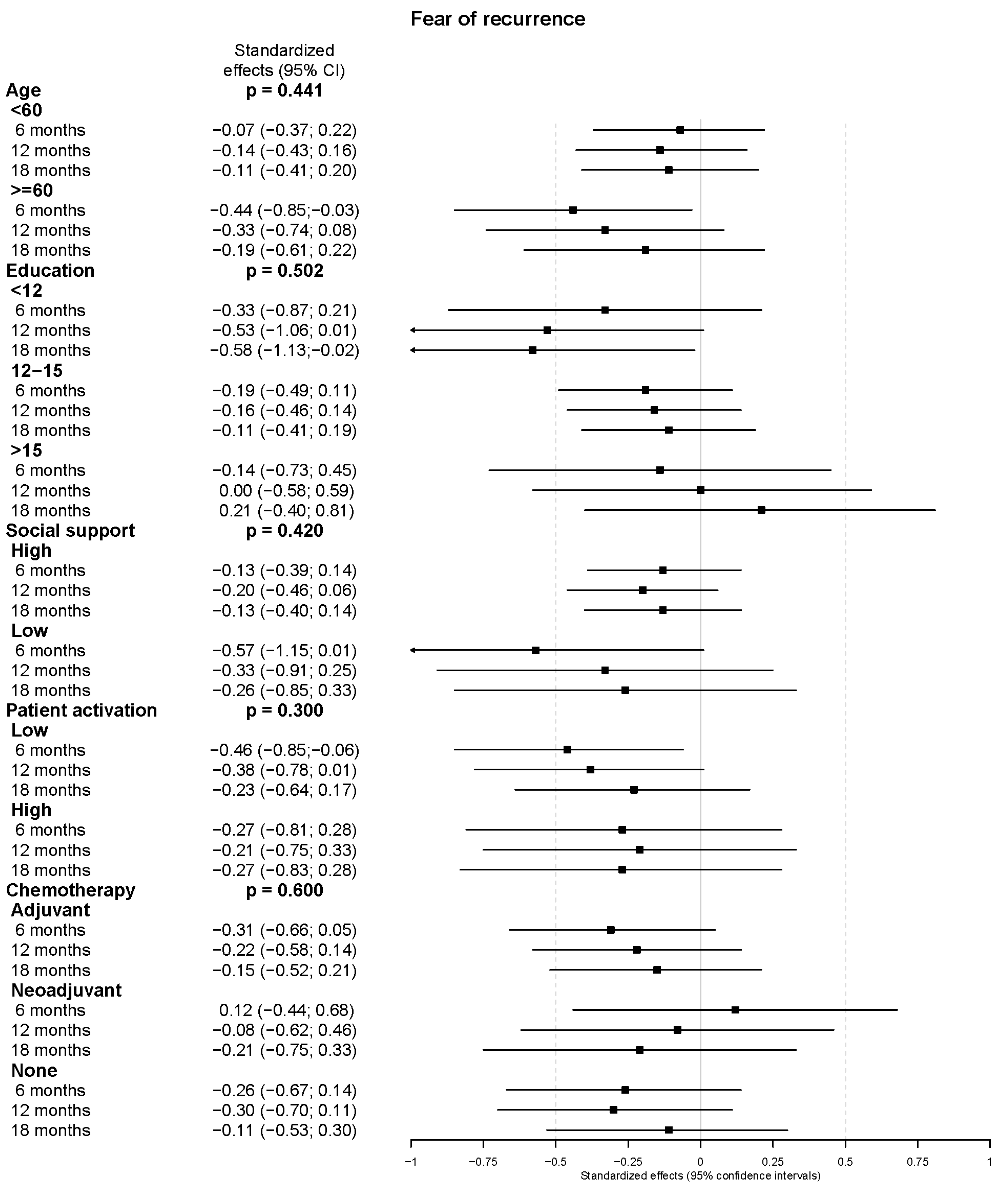

Note. Higher fear of recurrence score indicates worse fear of recurrence symptoms. Standardized outcome scores were applied using fitted models by subtracting the sample mean score from each score and dividing by the standard deviation. Models were adjusted for

© 2023 Bidstrup PE et al. *JAMA Network Open.*

randomization strata (age (< 60; ≥ 60 years) and treatment modality (no or adjuvant chemotherapy; neo-adjuvant).

© 2023 Bidstrup PE et al. *JAMA Network Open.*

eFigure 8. Standardized Interaction Effects for Patient Activation at 6, 12, and 18 Months Follow-up in 309 Patients With Breast Cancer in the REBECCA Study

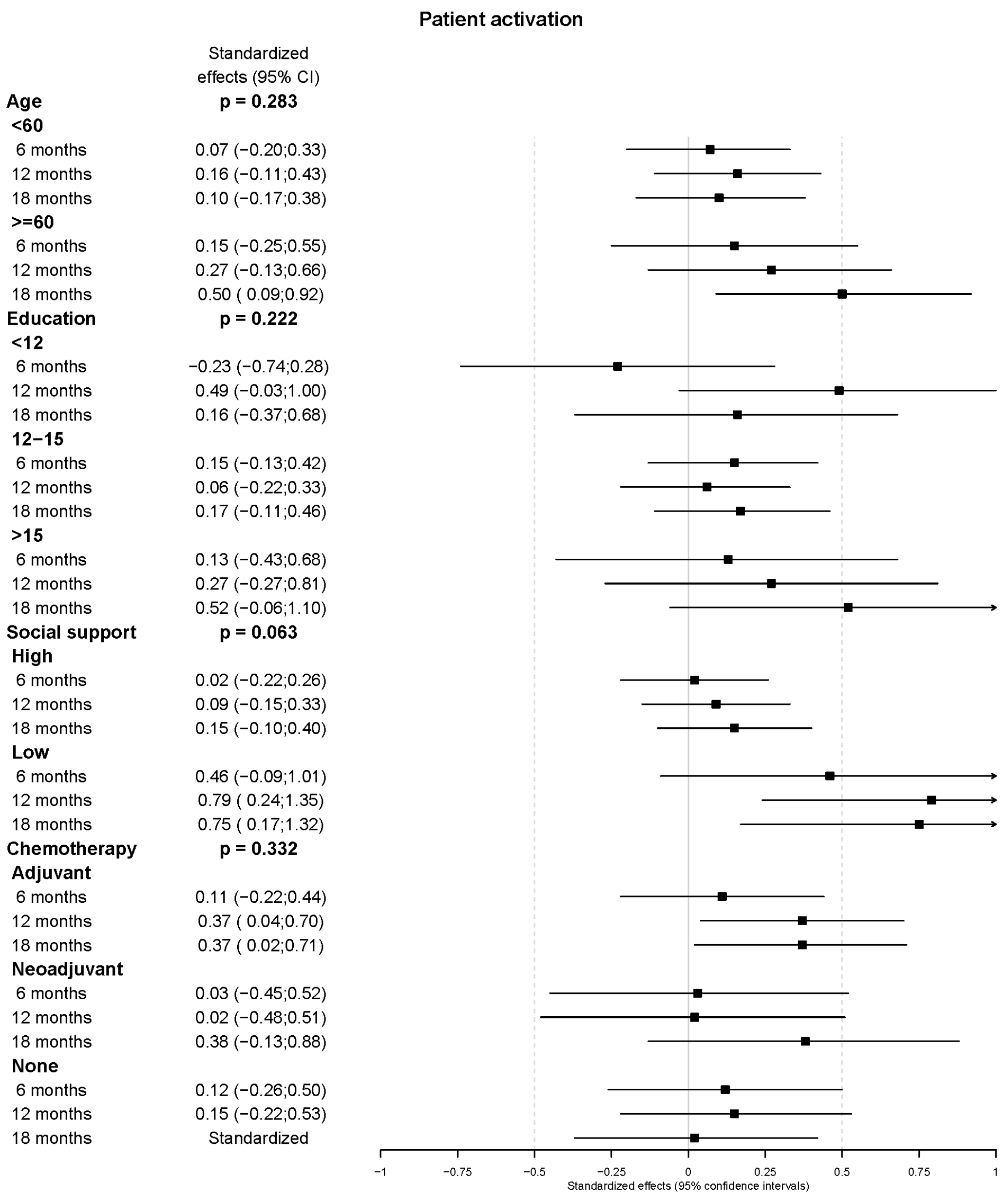

Note. Higher patient activation score indicates better activation. Standardized outcome scores were applied using fitted models by subtracting the sample mean score from each score and dividing by the standard deviation. Models were adjusted for randomization strata (age (< 60; ≥ 60 years) and treatment modality (no or adjuvant chemotherapy; neo-adjuvant).

© 2023 Bidstrup PE et al. *JAMA Network Open.*

eFigure 9. Standardized Interaction Effects for Cognitive Function at 6, 12, and 18 Months Follow-up in 309 Patients With Breast Cancer in the REBECCA Study

Cognitive function

	Standardized effects (95% CI)
**Age**	**p = 0.323**
**<60**	
6 months	0.08 (−0.13;0.30)
12 months	−0.03 (−0.25;0.20)
18 months	−0.03 (−0.25;0.20)
**>=60**	
6 months	0.27 (−0.03;0.57)
12 months	0.30 (0.00;0.61)
18 months	0.30 (−0.02;0.61)
**Education**	**p = 0.291**
**<12**	
6 months	−0.06 (−0.46;0.34)
12 months	−0.26 (−0.66;0.15)
18 months	0.05 (−0.38;0.48)
**12−15**	
6 months	0.22 (0.00;0.45)
12 months	0.26 (0.03;0.48)
18 months	0.18 (−0.05;0.41)
**>15**	
6 months	0.11 (−0.33;0.55)
12 months	−0.17 (−0.61;0.27)
18 months	−0.22 (−0.68;0.23)
**Social support**	**p = 0.122**
**High**	
6 months	0.10 (−0.10;0.29)
12 months	0.00 (−0.20;0.20)
18 months	0.03 (−0.17;0.24)
**Low**	
6 months	0.47 (0.04;0.90)
12 months	0.57 (0.14;1.00)
18 months	0.41 (−0.03;0.85)
**Patient activation**	**p = 0.443**
**Low**	
6 months	0.35 (0.04;0.66)
12 months	0.12 (−0.19;0.44)
18 months	0.04 (−0.28;0.36)
**High**	
6 months	0.11 (−0.32;0.54)
12 months	0.01 (−0.42;0.44)
18 months	0.12 (−0.33;0.56)
**Chemotherapy**	**p = 0.587**
**Adjuvant**	
6 months	0.15 (−0.11;0.41)
12 months	0.10 (−0.16;0.37)
18 months	0.04 (−0.24;0.31)
**Neoadjuvant**	
6 months	0.44 (0.05;0.83)
12 months	0.16 (−0.25;0.56)
18 months	0.23 (−0.17;0.63)
**None**	
6 months	0.02 (−0.28;0.32)
12 months	0.10 (−0.20;0.40)
18 months	0.14 (−0.17;0.45)

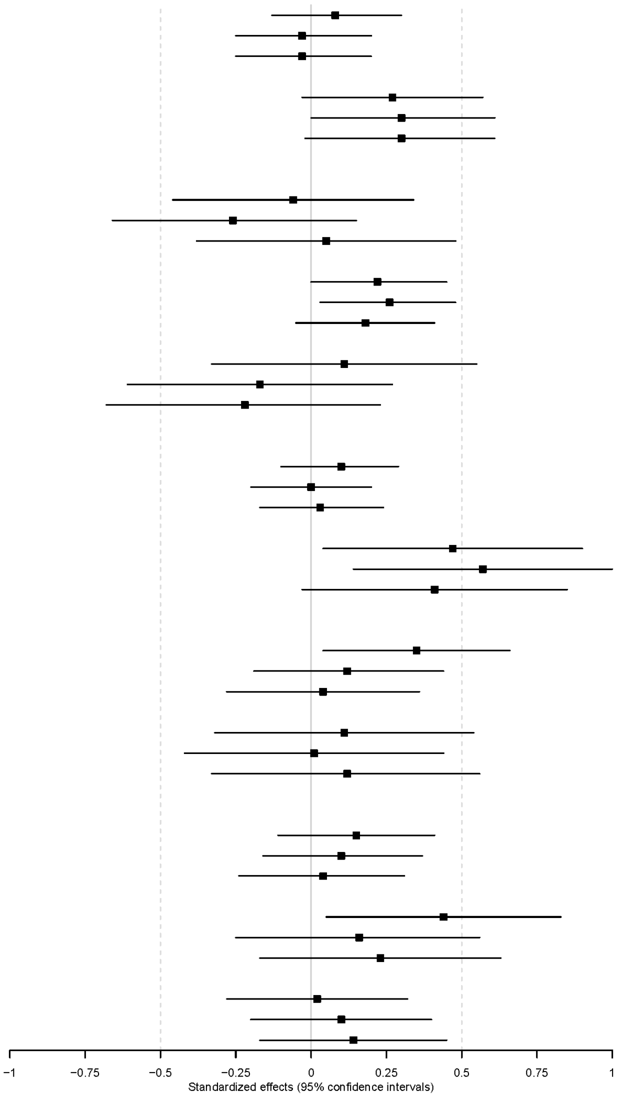

Note. Higher cognitive function score indicates better cognitive function. Standardized outcome scores were applied using fitted models by subtracting the sample mean score from each score and dividing by the standard deviation. Models were adjusted for randomization

© 2023 Bidstrup PE et al. *JAMA Network Open.*

strata (age (< 60; ≥ 60 years) and treatment modality (no or adjuvant chemotherapy; neo-adjuvant).

© 2023 Bidstrup PE et al. *JAMA Network Open.*
