# Supplementary material for: Effect of a Nurse Navigation Intervention on Mental Symptoms in Patients With Psychological Vulnerability and Breast Cancer: The REBECCA Randomized Clinical Trial
Source: JAMA Netw Open. 2023 Jun 23;6(6):e2319591. doi: 10.1001/jamanetworkopen.2023.19591 (PMC10290249; doi:10.1001/jamanetworkopen.2023.19591)
Supplement: Supplement 3. — Data Sharing Statement [file jamanetwopen-e2319591-s003.pdf]

Data Sharing Statement

Bidstrup. Effect of a Nurse Navigation Intervention on Mental Symptoms in Patients With Psychological Vulnerability and Breast Cancer. *JAMA Netw Open*. Published June 23, 2023. doi:10.1001/jamanetworkopen.2023.19591

Data
Data available: No

Additional Information
Explanation for why data not available: Data sharing is not possible due to European Union General Data Protection Regulation law where participants have to consent to each individual data sharing.
